# Characterization and simulation of metagenomic nanopore sequencing data with Meta-NanoSim

**DOI:** 10.1093/gigascience/giad013

**Published:** 2023-03-20

**Authors:** Chen Yang, Theodora Lo, Ka Ming Nip, Saber Hafezqorani, René L Warren, Inanc Birol

**Affiliations:** Canada's Michael Smith Genome Sciences Centre, BC Cancer, Vancouver, BC, V5Z 4S6, Canada; Bioinformatics Graduate Program, University of British Columbia, Genome Sciences Centre, BCCA 100-570 West 7th Avenue, Vancouver, BC, V5Z 4S6, Canada; Canada's Michael Smith Genome Sciences Centre, BC Cancer, Vancouver, BC, V5Z 4S6, Canada; Bioinformatics Graduate Program, University of British Columbia, Genome Sciences Centre, BCCA 100-570 West 7th Avenue, Vancouver, BC, V5Z 4S6, Canada; Canada's Michael Smith Genome Sciences Centre, BC Cancer, Vancouver, BC, V5Z 4S6, Canada; Bioinformatics Graduate Program, University of British Columbia, Genome Sciences Centre, BCCA 100-570 West 7th Avenue, Vancouver, BC, V5Z 4S6, Canada; Canada's Michael Smith Genome Sciences Centre, BC Cancer, Vancouver, BC, V5Z 4S6, Canada; Bioinformatics Graduate Program, University of British Columbia, Genome Sciences Centre, BCCA 100-570 West 7th Avenue, Vancouver, BC, V5Z 4S6, Canada; Canada's Michael Smith Genome Sciences Centre, BC Cancer, Vancouver, BC, V5Z 4S6, Canada; Canada's Michael Smith Genome Sciences Centre, BC Cancer, Vancouver, BC, V5Z 4S6, Canada; Department of Medical Genetics, University of British Columbia, Life Sciences Centre Room 1364 – 2350 Health Science Mall Vancouver, BC V6T 1Z3, Canada

**Keywords:** metagenomics, Oxford nanopore sequencing, microbial abundance estimation, sequence simulation, chimeric reads

## Abstract

**Background:**

Nanopore sequencing is crucial to metagenomic studies as its kilobase-long reads can contribute to resolving genomic structural differences among microbes. However, sequencing platform-specific challenges, including high base-call error rate, nonuniform read lengths, and the presence of chimeric artifacts, necessitate specifically designed analytical algorithms. The use of simulated datasets with characteristics that are true to the sequencing platform under evaluation is a cost-effective way to assess the performance of bioinformatics tools with the ground truth in a controlled environment.

**Results:**

Here, we present Meta-NanoSim, a fast and versatile utility that characterizes and simulates the unique properties of nanopore metagenomic reads. It improves upon state-of-the-art methods on microbial abundance estimation through a base-level quantification algorithm. Meta-NanoSim can simulate complex microbial communities composed of both linear and circular genomes and can stream reference genomes from online servers directly. Simulated datasets showed high congruence with experimental data in terms of read length, error profiles, and abundance levels. We demonstrate that Meta-NanoSim simulated data can facilitate the development of metagenomic algorithms and guide experimental design through a metagenome assembly benchmarking task.

**Conclusions:**

The Meta-NanoSim characterization module investigates read features, including chimeric information and abundance levels, while the simulation module simulates large and complex multisample microbial communities with different abundance profiles. All trained models and the software are freely accessible at GitHub: https://github.com/bcgsc/NanoSim.

## Background

Empowered by the rapid development of next-generation sequencing technologies, metagenomic analysis has enabled comprehensive investigation of the genetic composition and abundance of microbial communities. Metagenomic sequencing bypasses the need to culture each individual species by extracting DNA directly from their natural habitat, making it feasible to study microbes that cannot be isolated or cultured in the laboratory [1, 2]. Within the past few decades, the improved throughput and reduced cost of next-generation DNA/RNA sequencing platforms has enabled a wide range of metagenomic studies of environmental, pharmaceutical, and medical relevance [3–5].

Until recently, Illumina short-read sequencing (Illumina, Inc., San Diego, CA, USA) has been the technology of choice for metagenomic sequencing projects due to its high throughput, low cost, and low error rate. However, the reads generated by Illumina instruments are often too short (<250 bp) to span inter- and intrachromosomal homologous regions and suffer from intrinsic biases, thus complicating downstream assembly and taxonomic analysis [6]. As a third-generation long-read sequencing technology, nanopore sequencing from Oxford Nanopore Technologies Ltd. (ONT, Oxford, UK) is gaining traction in metagenomic research efforts, due largely to the long read lengths it generates, as well as the portability of some of their sequencing platforms [7]. ONT reads have highly variable lengths, ranging from tens to millions of base pairs (bp). For example, the N50 length (where 50% of bases sequenced are from reads of this length or longer) of ONT reads in a typical run is over 5 kbp [8], and the reported maximum read length exceeds 2 Mbp. At the high end, whole bacterial or viral genomes may be captured by a few sequencing reads [9, 10], making it possible to disambiguate between closely related strains. Since its introduction, ONT sequencing has played an essential role in real-time pathogen identification and clinical diagnosis, including research efforts during the COVID-19 pandemic [11–14].

Although a plethora of metagenomic analysis tools have been developed for short-read sequencing data, the challenges associated with ONT reads, such as high error rate, nonuniform error distributions, and chimeric read artifacts [8, 15–17], call for analytical tools designed specifically for long reads. For example, quantification of microbial abundance levels, or metagenomic abundance estimation, is traditionally computed by counting the number of mapped reads followed by fine-tuning of ambiguous mappings [18, 19]. This approach has been proven to be cost-effective for Illumina short reads because of their uniform lengths. However, the accuracy of these tools would be understandably impacted when applied on ONT reads, especially for lowly represented genomes, because of the variable lengths and relatively high error rates (5–15% depending on the flowcell chemistry and basecalling algorithm) compared to that of Illumina reads (typically less than 1%). In addition, ONT sequencing projects on genomes, transcriptomes, and metagenomes, from prokaryotes to eukaryotes, were all reported to contain certain problematic reads with gapped or chimeric alignments, likely generated due to library preparation or sequencing artifacts [17, 20–25]. Reference-based abundance estimation using merely primary alignments may further be affected by the presence of these chimeric reads, as well as reads that span the start position of a circular genome. To the best of our knowledge, even the state-of-the-art program, MetaMaps, does not account for chimeric reads but simply uses an expectation-maximization (EM) algorithm to disambiguate multimapped reads [26]. In this work, we show that there is still room for improving metagenomic abundance estimation, a proposition attainable by quantifying aligned bases instead of reads, while leveraging chimeric read information.

In the process of tool development and benchmarking, a metagenomic ONT read simulator and associated simulated datasets with known ground truth can save time and money. Ideally, such a read simulator should reflect the true characteristics of the ONT platform and allow effective evaluation of bioinformatics tools. In return, the evaluation results can guide the experimental design of metagenomics projects, to help determine the desired sequencing depths and number of replicates [27].

Currently, the only simulator that specifically simulates ONT metagenomic datasets is CAMISIM [28]. The workflow of CAMISIM is focused on the composition design of a microbial community given a taxonomy profile, while the abundance levels are drawn from a lognormal distribution. The obvious drawback of this approach is that users cannot request the abundance levels as they need. CAMISIM uses NanoSim [15] as its engine to simulate ONT reads for each genome separately once the composition of the community is determined. Following the same idea, one can also use other existing ONT genomic simulators naively to simulate each composite genome separately and then aggregate the reads according to the desired abundance. However, it is impractical to simulate a large microbial community with hundreds or more genomes with this approach, not to mention that the existing simulators for ONT reads are not designed to model metagenomic-specific features, such as chimeric reads and deviations in abundance levels. More important, the simulation of abundance levels should be consistent with the quantification method, and thus merely mixing the reads from different genomes will yield a compromised abundance profile. Taken together, we note that the previous version of NanoSim can be upgraded to capture and simulate read properties specific to metagenomics, especially the microbial abundance levels and chimeric reads—2 key factors that may influence metagenome assembly, taxonomy binning, and abundance estimation. Further, in real-world scenarios, viruses, bacteria, and fungi coexist in complex microbial communities; hence, the ability to simulate complex metagenomes comprising both circular and linear genomes is very important.

Here, we introduce Meta-NanoSim (released within NanoSim version 3), an ONT metagenome simulator for complex microbial communities. Given a training dataset, Meta-NanoSim characterizes read length distributions, error profiles, and alignment ratio models. Optionally, it also detects chimeric reads and estimates microbial abundance levels. In our benchmarks, the performance of the metagenomic abundance estimation feature of Meta-NanoSim surpasses the current state-of-the-art methods. The chimeric read detection feature also improves the read length modeling, and thus simulating this artifact of the technology may challenge metagenomic analytical tools with a real-world scenario. Through benchmarking experiments comparing simulated reads with empirical datasets, we show that Meta-NanoSim preserves the key characteristics of ONT metagenomic reads. Finally, we showcase the usability and utility of Meta-NanoSim in assessing the performance of a metagenomic assembly tool.

## Implementation

### Meta-NanoSim general design

**Figure 1:**
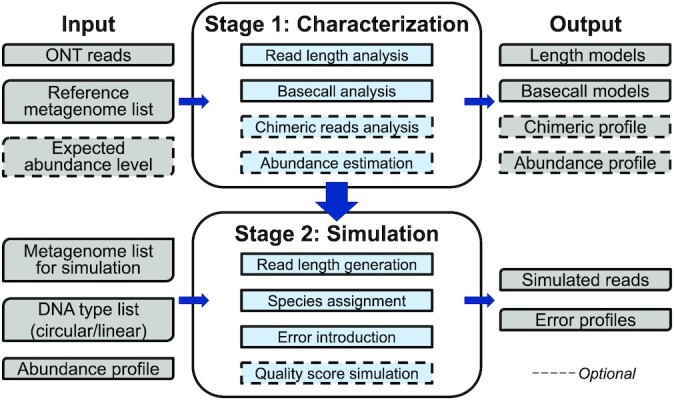
Meta-NanoSim workflow. Meta-NanoSim consists of 2 stages: characterization and simulation. In the characterization stage, given a training dataset and reference metagenome, Meta-NanoSim builds models for the read length distributions and basecall events. It optionally profiles chimeric read artifacts and quantifies an abundance profile. It can also calculate the deviation between expected and estimated abundance levels. In the simulation stage, Meta-NanoSim takes 4 inputs: (i) a list of genomes to be simulated, (ii) a list of genome topologies, (iii) target abundance profiles, and (iv) the models generated from the characterization stage. Meta-NanoSim outputs simulated reads and error profiles.

Meta-NanoSim is implemented in Python as the “meta” submodule for both characterization and simulation stages within the NanoSim suite. It learns the technical and metagenomic-specific features of ONT reads in the characterization stage, builds statistical models, and applies them in the simulation stage (Fig. 1). In the characterization stage, it takes ONT metagenomic reads and a reference metagenome as input to infer the ground truth through sequence alignments. Based on those alignments, the read length distributions (for aligned and unaligned reads) and basecall events are modeled via kernel density estimation and mixture statistical models, respectively. In addition to existing NanoSim features, we introduce 2 new analyses in the characterization stage: chimeric read analysis for genome/metagenomes and abundance estimation for metagenomic datasets.

Simulating a metagenomic dataset with Meta-NanoSim requires 4 inputs: (i) a list of reference genomes to be simulated (local file paths or web addresses), (ii) target abundance levels, (iii) genome topologies (i.e., linear or circular), and (iv) the pretrained model from the characterization stage. Meta-NanoSim can optionally stream reference genome sequences from either RefSeq [29] or Ensembl [30] automatically without requiring extra disk storage, which facilitates large microbial community simulations. Since microbial sequencing projects are often carried out in a multisample or multireplicate fashion, Meta-Nanosim is designed to simulate multiple samples in 1 batch with user-defined abundance-level profiles as input.

### Chimeric read detection and simulation

A chimeric read, also known as a “split read,” has 2 or more subalignments against distinct regions of the reference genome/metagenome. They may arise because of sequencing artifacts or may appear like structural variants when the source genome is absent from the reference metagenome. More specifically, sequencing reads may be mapped to a similar but structurally different genome (e.g., a different strain or subspecies), causing segments of the read to align to different regions of the reference genome. When the query and reference coordinates of subalignments do not overlap, we define them as a set of compatible alignments. Finding the best compatible alignment set problem is akin to the interval scheduling maximization problem, which finds a set of nonoverlapping intervals of maximum size. For each read, we exhaustively search for all compatible alignments for each subalignment to generate a list of compatible alignment sets (Supplementary Fig. S1). We then select the best element from the list for downstream analysis, based on alignment quality and total aligned length. If, for a given read, the best element contains 2 or more compatible alignments, the read is considered chimeric, and its aligned length, gap length, and source species (specific to metagenome mode) are modeled for simulation. Exceptions are reads bridging the start and end of a circular genomic reference; these reads are detected but not designated as chimeric, and the subalignments within these reads are concatenated as single alignments.

To determine the source species for each segment in chimeric reads, we built a simplified hidden Markov model where the start probability is the input abundance, the emission probability represents which species the next segment is coming from given the previous one, and the transitional probability of species is the change of abundance in the underlying Markov chain. Since the species to be simulated—namely, the states in a Markov model—may be different between the training and simulation metagenome, we generalize the emission probability as a single value called shrinkage rate *s* (0 < *s* < = 1). This parameter describes the reduction of abundances (probabilities) of other species, while maintaining the relative abundances among them. Assuming the input abundance is {*p_A_, p_B_, p_C_, …, p_N_*} for *n* species, when the first segment comes from species A, the transitional probabilities for the other species would become {*s*$\times $*p_B_, s*$\times $*p_C_ … s*$\times $*p_N_*} and the transitional probability for A would be inflated as 1–*s*$\times \mathop \sum \limits_{i\ = \ B}^N {p}_i$. To learn *s*, all segments in chimeric reads are divided into overlapping pairs, and the probability for the source species of the second segment being different from the first one is recorded. In this way, we can calculate the reduction of abundance for every species. The average reduction is the shrinkage rate, and the inflated abundance for being from the same species can be inferred as well. The shrinkage rate can also be adjusted by the user—to 1, for example—if one assumes all DNA molecules are homogeneously suspended in the buffer.

In summary, Meta-NanoSim first determines the number of segments to be simulated based on a geometric distribution. A read is chimeric if it has 2 or more segments. Then, Meta-NanoSim generates the lengths of each segment and the gap(s) between them using kernel density estimation learned from empirical reads. The source species of the first segment is randomly picked based on the input abundance level. Starting from the second segment, the abundance levels are recomputed based on the previous species and *s*. The source species is determined one after another, and then sequences are extracted, mutated with purposely introduced errors, and collated in the same process as nonchimeric reads.

### Abundance estimation

Existing abundance estimation methods generally quantify the number of mapped reads or *k*-mers, under the presumption that all reads have equal lengths. However, since the ONT read length varies across several orders of magnitude, the mean read length for each species is likely to be different. When all species are equally and deeply sequenced, according to the central limit theorem, the standard deviation of mean lengths would scale with $1/\sqrt n $, where *n* is the number of species. For finite data, low-abundance species may have a higher standard deviation because there are fewer sequences representing them. We observe the mean read lengths of uniquely aligned reads to vary substantially in datasets where species abundance levels are logarithmically distributed, necessitating base-level instead of read-level quantification algorithms (Supplementary Fig. S2).

Another key challenge that confounds short-read metagenomic analysis is ambiguously aligned reads. In ONT datasets, however, most reads are long enough to span inter- and intraspecies homologous regions, and the chimeric read detection feature can resolve the estimation for reads having multiple subalignments and for reads bridging the start and end of a circular genome. For the remaining small fraction of multialigned reads between closely related species, the estimation for them can be optimized using the EM algorithm.

The EM algorithm (Algorithm 1) first processes uniquely aligned reads to calculate a baseline abundance profile. Then it starts the expectation step, which is to assign multialigned bases proportionally to their respective species based on their relative abundances. Next, in the maximization step, these multialigned bases are used to update the abundance profile. The algorithm then goes back to the expectation step to update the fractions of multialigned bases based on the new abundance profile. The E and M steps alternate until the difference in abundances between 2 rounds is lower than a threshold (default: 1%). Note that the abundance levels are in the units of relative genomic DNA weight, and they can be used to calculate genome copy numbers when divided by the respective genome sizes.

**Table:** 

**Algorithm 1** EM for metagenome abundance estimation
abundance_list = {species1: abundance1; species2: abundance2; …}
base_count = {species1: count1; species2: count2; …}
Start processing uniquely aligned reads:
for each uniquely aligned read and its source species:
base_count[species] + = aligned bases
abundance_list = {species: base_count[species]/sum(base_count[species])}
Start processing multialigned reads
while diff > = min(abundance_list.values()) * 0.01:
**E-Step:**
for each multialigned read:
read_abun = the sum of abundances for all possible species for that read
for each possible species:
fraction = aligned bases * abundance_list[species]/read_abun
base_count[species] + = fraction
**M-Step:**
abundance_list = {species: base_count[species]/sum(base_count[species])}
diff = |abundance_list—prev_abundance_list|

Meta-NanoSim offers abundance estimation with or without chimeric read detection. When chimeric read detection is enabled, all subalignments are used for computing estimates; otherwise, only primary alignments are used. Meta-NanoSim records the aligned bases for each subalignment toward their source genome and then uses the EM algorithm to assign multialigned segments proportionally to their putative source genomes iteratively.

### Abundance deviation simulation

Meta-NanoSim simulates abundance deviation with user-defined lower and upper deviation boundaries. We noticed a weak positive correlation between genome size and abundance deviation in our analysis. During simulation, we first randomly draw a list of relative error between the deviation boundaries. We then assign these errors to each genome based on their sizes—namely, larger deviations are assigned to larger genomes and smaller ones are assigned to smaller genomes. Finally, abundance values are normalized for a total abundance of 100%.

## Results

We first assess the performance of the 2 key features in Meta-NanoSim, chimeric read detection and abundance estimation. To evaluate the similarity between simulated reads and experimental reads, we generated 2 simulated datasets using models learned from experimental data, and we compared the performance of Meta-NanoSim with that of CAMISIM. We illustrate that Meta-NanoSim is capable of simulating a large complex microbial community containing 125 species based on a human saliva sample. Finally, we showcase an application of Meta-NanoSim simulated data in benchmarking the long-read metagenomic assembler MetaFlye [31]. Specifically, we evaluated the assembly quality and scalability of MetaFlye with respect to increasing sequencing depths.

### Evaluation of chimeric read characterization and simulation

**Figure 2:**
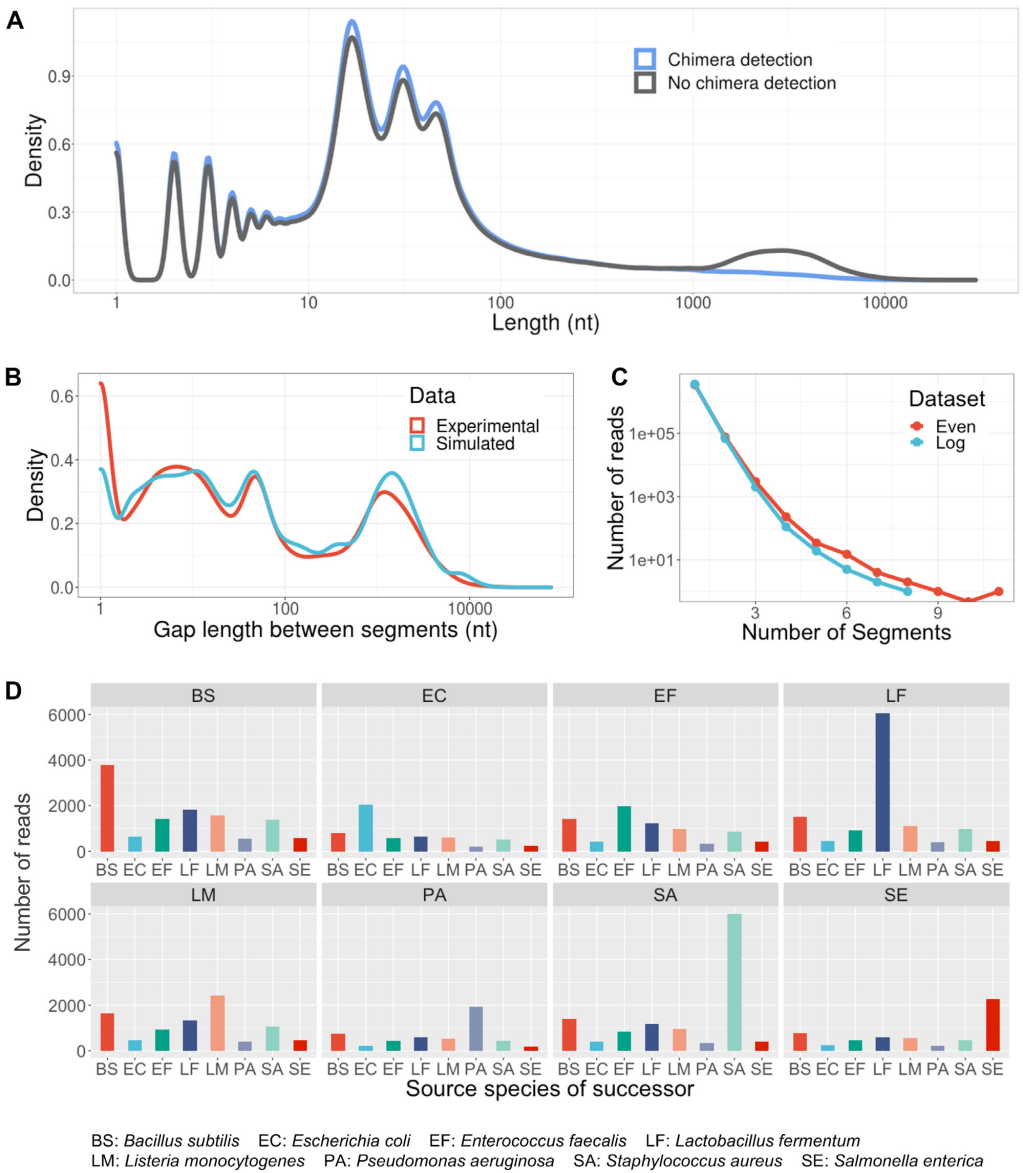
Evaluation of chimeric read detection and simulation. (A) The length distribution of the unaligned regions of reads with or without chimeric read detection for the *Log* dataset (x-axis in logarithmic scale). (B) The performance of gap length simulation for the *Log* dataset (x-axis in logarithmic scale). (C) The number of segments each read contains for the *Even* and *Log* datasets. (D) All segments in chimeric reads in the *Even* dataset are converted into overlapping pairs. Each facet represents 1 source species of the first segment, and the x-axis represents the source species of the second segment. Each facet shows the probability of the second segment given the source species of the first one. *Cryptococcus neoformans* and *Saccharomyces cerevisiae* are excluded here due to their low abundances.

Previous studies reported that chimeric reads represent a nonnegligible fraction of ONT sequencing datasets ranging from 1.70% to 8.17% depending on the sequencing kits and identification thresholds [17, 20, 22, 23]. In the metagenome datasets used in our study, after ruling out structural variants, we have identified a similar fraction of reads in this category: 2.17% (75,628 reads) in the *Even* dataset and 1.67% (68,444 reads) in the *Log* datasets. These reads are free of known adapters, so their presence may impact downstream analyses, such as assembly, taxonomy binning, and quantification, even after adapter trimming. When aligned to their respective reference genome sequence(s), ONT reads may contain unaligned or soft-clipped regions. In our tests, the chimeric read detection feature of Meta-NanoSim significantly reduced the length of these unaligned regions, which explained why some of the reads have over 1-kbp-long unaligned portions (Fig. 2A). As seen in Fig. 2B, the length distributions of the gaps between split alignments follow multimodal distributions. Meta-NanoSim uses kernel density estimation to model them, with results exhibiting strong similarity between the length distributions of simulated and experimental sequences. We also noticed that the number of segments each read contains can be described as a geometric distribution, and the mean probability can be learned from experimental data (Fig. 2C). On average, each read contains 1.03 segments for both data sets under study.

Based on the source species to which each split alignment belongs, chimeric reads can be classified as “intraspecies chimeric” or “interspecies chimeric.” It is observed that the source species of the first segment is affected by the abundance level, while the subsequent segment is more likely to be influenced by the identity of the previous species (Fig. 2D). We postulate that this is because DNA molecules of the same species are more likely to gather near the nanopore than being homogeneously dispersed in the buffer. Regardless of the actual cause, this phenomenon can be approximated as a simplified hidden Markov model with a generalized emission probability, which is defined as shrinkage rate *s* here. To our calculation, *s* is equal to 0.77 for the *Even* dataset and 0.73 for the *Log* dataset, suggesting that its value may be stable across datasets.

### Evaluation of abundance estimation

We compared the performance of 4 abundance estimation methods: Meta-NanoSim estimation with chimeric read detection, Salmon quantification with the “—meta” option (Salmon) [32], the base-level estimation reported in the paper that released the dataset (denoted as “Data Note” from here on) [8], and MetaMaps. For Meta-NanoSim estimation, we performed an ablation study that removes key components of the algorithm step by step, including estimation on read level with chimeric read detection (Meta-NanoSim CR) or with the EM algorithm (Meta-NanoSim ER), estimation on base level (Meta-NanoSim B), base level with chimeric read detection (Meta-NanoSim CB), base level with the EM algorithm (Meta-NanoSim EB), and base level with chimeric read detection fine-tuned by the EM algorithm (Meta-NanoSim ECB). All compared methods, except for MetaMaps, are computed based on Minimap2 alignments. We compared the estimated abundances to the expected values provided by the manufacturer based on *R*-squared, standard deviation, and percent error.

In general, all base-level quantification methods (i.e., Meta-NanoSim B, CB, EB, and ECB and Data Note) performed better than read-level quantification methods (i.e., Salmon, MetaMaps, and Meta-NanoSim CR and ER), and Meta-NanoSim base-level estimations have the highest correlation with the expected abundances (Table 1, Supplementary Fig. S3). For the *Even* dataset, all 4 Meta-NanoSim base-level methods performed similarly; the stand-alone base-level quantification has the highest *R*-squared value for the *Even* dataset, while the chimeric read detection helped reduce the percent error, mainly for low-abundance species *Cryptococcus neoformans* (Supplementary Fig. S3). MetaMaps, as a read-level quantification method designed specifically for ONT metagenomic data, although ranked highest among this category, has twice the percent error than than base-level methods. For the *Log* dataset, Meta-NanoSim base-level estimations also had similar *R*-squared values, higher than other methods. Although the metrics are very similar for the *Log* dataset, the performance on low-abundance species may be overshadowed by high-abundance species. Therefore, we computed the coefficient of correlation and error between log-transformed estimated and expected abundances. After log-transformation, Salmon, Meta-NanoSim ECB, and Meta-NanoSim EB performed similarly; Salmon has the highest correlation and Meta-NanoSim ECB has the lowest percent error. The metrics for Meta-NanoSim estimation without EM, on the other hand, decreased significantly due to difficulty in differentiating multimapped reads for low-abundance species. When estimating the abundance levels for the *Log* dataset, Minimap2 incorrectly assigned 18,212 reads to the *Enterococcusfaecalis* genome as primary alignments, but these reads can also align to an interspecies homologous region in the *Listeria monocytogenes* genome. In fact, *E. faecalis* is a low-abundance species in the *Log* dataset with only 33 unique alignments. Therefore, the methods with the EM algorithm resolved the multialigned reads problem, indicating that EM can be advantageous for datasets consisting of similar genomes but with large variances in abundance levels.

**Table 1:** Statistical analysis of the abundance estimation results compared to expected abundances

Tool	Algorithm				*Even* dataset			*Log* dataset			
	E	C	B	R	*R* ^2^	SD	PE	*R* ^2^	Log *R*^2*^	SD*	PE
**Meta-NanoSim**	√	√	√		0.7463	0.0225	144.5317	**1.0000**	0.9920	0.1818	**256.7856**
		√	√		0.7465	0.0225	**144.0877**	0.9999	0.7899	0.9295	53,349.95
	√		√		0.7498	**0.0224**	145.6229	**1.0000**	0.9917	0.1843	260.6973
			√		**0.7499**	**0.0224**	145.4656	**1.0000**	0.7895	0.9304	53,443.94
		√		√	0.4305	0.0337	326.3432	0.9980	0.7778	0.9560	57,527.07
	√			√	0.4396	0.0335	313.1466	0.9980	0.7776	0.9565	57,651.72
**Salmon**	√			√	0.4269	0.0339	318.1502	0.9978	**0.9955**	**0.1366**	261.1368
**Data Note**			√		0.6702	0.0257	181.1667	0.9998	0.9863	0.2374	359.9314
**MetaMaps**	√			√	0.4420	0.0334	258.4563	0.9979	0.9652	0.3781	1,366.705

B, base-level quantification; C, chimeric read detection; E, EM algorithm; PE, summation of percent error; R, read-level quantification. The best values for each column are in bold.

* The expected and estimated abundances are log-transformed before calculating the *R*-squared value and standard deviation.

To recapitulate our findings, we utilized another logarithmically distributed mock microbial community from a previous study [33] (denoted as the *Adp* dataset from here on) and repeated the quantifications with Meta-NanoSim base-level methods, Salmon, and MetaMaps (Supplementary Table S1). As we had previously observed, all 4 Meta-NanoSim methods performed similar to each other with the highest correlation to the expected values. MetaMaps quantification, although with the lowest percent error among all compared methods, showed a much lower correlation in terms of *R*-squared and standard deviation. Taken all together, Meta-NanoSim base-level quantification, after chimeric read detection and fine-tuning by the EM algorithm, had balanced correlation and percent error robustly, thus making it preferable for naturally occurring microbial communities with varying abundance levels.

**Figure 3:**
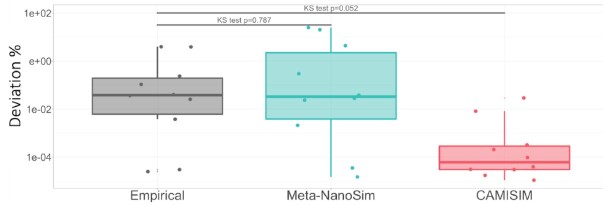
Abundance-level deviations between experimental and simulated metagenomic reads. In this plot, each dot represents a microbial genome, and the y-axis represents the deviation in percentage between the expected values and experimental/simulated values.

Because of the deviation between expected and estimated abundance levels, we introduce a feature that can simulate this observation. We compared the deviation between expected abundances and experimental data, as well as the simulation results of NanoSim and CAMISIM (Fig. 3). The distribution of abundance deviations for experimental data and Meta-NanoSim simulated reads is statistically the same (Kolmogorov–Smirnov test *P* = 0.787), while the distributions for CAMISIM simulated reads and experimental data are noticeably inconsistent, as CAMISIM does not provide this feature.

### Comparison between simulated and experimental datasets

To demonstrate the performance of Meta-NanoSim, we trained it with the *Log* dataset and compared the simulated datasets against the result of CAMISIM. With 8 processors, simulation of 1 million reads took under 20 minutes (or under 160 CPU-minutes) for Meta-NanoSim, while CAMISIM required more than 6 hours to complete.

**Figure 4:**
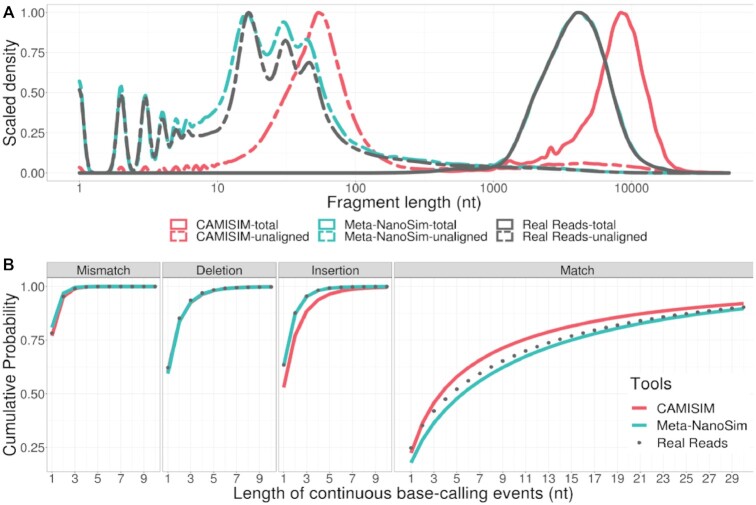
Performance of Meta-NanoSim and CAMISIM in simulating 1 million reads from the *Log* dataset. (A) Comparison of read length distributions in the empirical vs. simulated reads (x-axis in logarithmic scale). Unaligned length represents the length of unaligned part of each aligned read. (B) Cumulative probability distributions of the lengths of matches/errors in empirical and simulated reads.

The read lengths of simulated datasets from Meta-NanoSim follow the empirical length distribution closely, with a median read length peak at 4,040 nt (3,994 nt for empirical reads) (Fig. 4A). In contrast, the lengths of CAMISIM simulated reads deviate far from those of the empirical data. Moreover, the length distribution of unaligned regions on Meta-NanoSim simulated reads captures the patterns in empirical reads well, with multiple peaks below 100 nt. In contrast, the lengths of the unaligned part in CAMISIM reads are inflated as it does not detect or simulate chimeric reads. Both Meta-NanoSim and CAMISIM mimic the mismatch and deletion events well when compared to the empirical dataset (Fig. 4B), which demonstrates the robustness of NanoSim mixture statistical models. However, Meta-NanoSim simulates insertion and match events better than CAMISIM, also due to the change of model.

Additionally, we challenged Meta-NanoSim with 2 simulation tasks to mimic real-world use cases. First, we simulated 2 samples at the same time with the pretrained model from the *Log* dataset. Each sample contained 1 million reads from the 7 species from the *Adp* dataset with different abundance levels. Meta-NanoSim simulation finished within 51 minutes with 8 processors. Although the metagenome to be simulated is completely different from the one used for training, simulated reads exhibited similar read features as the experimental data, and the abundance levels are highly in accordance with the expected values (Supplementary Figs. S3 and S4). Next, we randomly picked a saliva sample from the Human Microbiome Project and simulated ONT reads using the same microbial composition [34]. The abundance levels of the 125 different bacteria strains range between 4.28% and 12.49%. By streaming reference genomes from RefSeq directly, it took Meta-NanoSim less than 3 hours to simulate 10 million reads, including the time used for streaming reference genomes from the RefSeq server.

### Application in metagenome assembly benchmarking

To demonstrate the utility of Meta-NanoSim, we simulated 4 sets of data with 1, 2, 4, and 10 million reads (denoted as “1M,” “2M,” “4M,” and “10M” from here on, respectively) based on models learned from the *Log* dataset to assess the correctness, scalability, and robustness of metaFlye. For the sake of simplicity and uniformity, all simulated reads presented herein were generated in FASTA format. We note that Meta-NanoSim can optionally simulate quality scores for reads in FASTQ format (Supplementary Fig. S5). With 128 threads, runtimes ranged from 1 hour to 7 hours (Supplementary Table S2). The maximum resident set size for the 10M dataset was 212 GB, but intermediate files occupied over 10 TB of disk space during the consensus-building stage. We also tried to assemble a larger dataset of 20 million reads, but the assembly failed after 30 days with an out-of-memory error on a 1-TB RAM server. According to the log file, the graph simplification was the most time-consuming stage, which lasted for 2 weeks.

**Figure 5:**
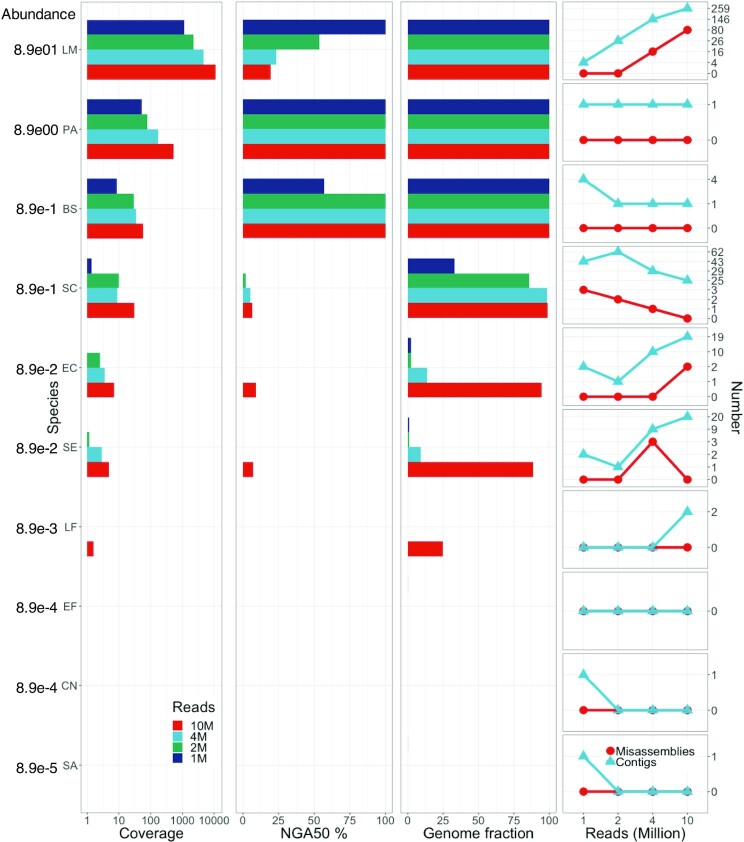
metaFlye assemblies with 4 sets of simulated metagenome sequencing data. The 4 sets of simulated datasets include 1, 2, 4 and 10 million reads, respectively. The abundance is the expected abundance level during simulation. The coverage panel shows the average read depth, including plasmids (x-axis in logarithmic scale). NGA50% represents the NGA50 length divided by the reference genome size. Genome fraction is a proportion between the assembled sequences and each corresponding genome. The right-most panel shows the number of misassemblies and assembled contigs as the number of simulated reads increases. BS, *Bacillus subtilis*; CN, *Cryptococcus neoformans*; EC, *Escherichia coli*; EF, *Enterococcus faecalis*; LF, *Lactobacillus fermentum*; LM, *Listeria monocytogenes*; PA, *Pseudomonas aeruginosa*; SA, *Staphylococcus aureus*; SC, *Saccharomyces cerevisiae*; SE, *Salmonella enterica*.

For the 4M dataset, the MetaFlye assembly has a total reconstruction of 27.81 Mbp, which is equivalent to 43.72% base coverage of the reference metagenome. These metrics are similar to the reported assemblies using the original training dataset with 3.48 million reads (28.20 Mbp assembled length that covered 46.0% of the reference metagenome) [31]. The average fold coverage is positively correlated to the number of sequencing reads and abundance levels, and accordingly, the genome reconstruction fraction and NGA50 length are positively correlated to the average fold coverage (Fig. 5). As expected, genomes with less than 1× coverage have very poor reconstructions. Between 1× and 10× coverage, the positive correlation is mirrored in multiple species, including *Bacillus subtilis, Saccharomyces cerevisiae, Escherichia coli*, and *Salmonella enterica*. When the coverage reaches 10×, metaFlye is able to reconstruct the genome to nearly 100% (*S. cerevisiae* in the 4M dataset, Fig. 5). When the coverage reaches 30×, the NGA50 length can cover the whole genome size (*B. subtilis* in the 2M dataset). Similarly, the number of contigs reconstructed for these 2 species decreases as the number of reads increases, showing how increasing sequencing depth can help assemble genomes into 1 contig for *B. subtilis* and nearly 1 contig per chromosome for *S. cerevisiae*. In contrast, when there is insufficient coverage, the assembled genome may be fragmented or even misassembled, as in the case of *E. coli* and *S. enterica*. However, a higher coverage does not necessarily lead to a better assembly quality. The reconstruction of *L. monocytogenes* deteriorates with more reads when the fold coverage exceeds 1,000×. Although the genome fraction remains 100%, the NGA50 length is only half or less of the genome size for the 2M, 4M, and 10M datasets. The drop in NGA50 length can be explained by the increasing number of reconstructed contigs and misassemblies (Fig. 5). With 1 million reads, only 4 contigs can be mapped to the *L. monocytogenes* genome, and no misassemblies were detected. However, we think that the fold coverage above 1,000× has led to many more misassemblies, adversely affecting the assembly contiguity as measured by the NGA50 length metric.

## Discussion

The applications of nanopore sequencing on metagenomic projects are rapidly expanding, motivating the development of metagenomic analysis tools tailored for this specific data type. In this work, we have introduced 2 main contributions to ONT metagenomic analysis tasks: (i) a new base-level quantification method for metagenomic abundance estimation and (ii) an upgrade of NanoSim for metagenomic characterization and simulation.

Reference-based metagenomic abundance estimation is key to investigating the microbial composition of an environment, enabled by emerging sequencing technologies. The long read length of ONT reads provides an opportunity to resolve homologous regions between species or strains, but complications arise due to their high error rates and nonuniform read lengths. Existing methods, primarily developed for short-read technologies, generally assume uniform read lengths and therefore only need to count the number of mapped reads or *k*-mers. For example, a 100-bp read and a 10,000-bp read do not have an equal contribution to the genome abundance. We have shown that it is necessary to quantify microbial abundances on a base level rather than a read level to better leverage this data type. In addition to their higher error rates, a small yet substantial fraction of ONT reads are chimeras, which may obscure the accuracy of estimates. The chimeric read detection feature in Meta-NanoSim searches for best compatible alignments and reduces the percent error in microbial abundance estimation. We adopted an EM algorithm to optimize the proportional contributions of ambiguous multialigned segments to each potential source species. From our benchmarking results, we demonstrated that the combination of these three components can improve correlations with expected abundances. We note that Meta-NanoSim quantification performs better when the abundance levels are more uniform or when low-abundance microbes do not share large homologous regions with high-abundance microbes. Depending on whether the user wishes to achieve a higher correlation or lower percent error, they can choose to disable or enable chimeric read detection, respectively. Although our work is limited to reference-based quantification, we expect it to inspire the design of reference-free methods and eventually have a broader application.

Built on top of abundance estimation, Meta-NanoSim is able to simulate datasets with desired abundance profiles. It can also recapitulate the abundance-level deviation from expected values, an especially useful feature for designing sequencing projects. When the abundance of a microbial community is known (or estimated), it is essential to determine the sequencing depth that ensures sufficient representation of each species. However, when the sequenced abundance differs from the expected value, simulated data with abundance variations true to the platform can inform the relationship between sequencing depth and abundance levels.

The general workflow of characterization and simulation of Meta-NanoSim follow the same paradigm of the previous versions of NanoSim. The chimeric read detection in the characterization stage provides a means to profile all chimeric reads in a library regardless of its root cause. When the reference metagenome is inclusive, the chimeric reads are likely introduced by library preparation and sequencing artifacts, while in reality, since the detection relies on alignment, some chimeric reads may also be attributed to structural variants when the source genome is not present in the reference. In this case, the output of the characterization stage can be used to further investigate such events with specifically designed statistical models and algorithms.

The 3 new main features added to Meta-NanoSim are (i) chimeric read simulation, (ii) the ability to stream reference genomes from online servers, and (iii) the simulation of a metagenome composed of a mixture of both linear and circular genomes. As chimeric reads may interfere with downstream analyses, simulated datasets with these artifacts are needed for more accurate performance assessment. Characterizing this feature and introducing it to simulated reads will also diversify error types in the reads, helping to improve the robustness of related algorithms. Reference genome streaming is uniquely advantageous when simulating a large metagenome with hundreds of species. It is a convenient alternative to manual file downloads of reference genomes, and it saves disk space while keeping the runtime reasonable. Similarly, since metagenomes are naturally composed of both linear and circular genomes, having a simulated dataset supporting this important characteristic will add credibility to benchmarking results and better forecast performance with experimental data.

The benchmarking on a metagenome assembly task showcased that Meta-NanoSim can facilitate relevant tool development as well as guide sequencing projects. The resulting assembly quality of Meta-NanoSim simulated reads is comparable to that of the experimental data with similar coverage. Although publicly available mock community sequencing data provide a more realistic training and test set, simulated data provide a ground truth and have virtually no limit in size, making them perfect for testing the accuracy and scalability of algorithms. Through the use of simulated datasets, we demonstrated that metaFlye assembler performs best when the species coverage is between 10× and 1,000×. To ensure a successful assembly of low-abundance species, it is suggested to calculate the number of reads needed given an estimated abundance first to ensure just enough coverage without wasting resources. For example, it takes 10 million reads to achieve 10-fold coverage for a 0.1% abundance species with a genome size of 5 Mbp. When assembling real microbial communities with highly variable abundance levels, we recommend multiple rounds of assembly with different sample sizes to achieve the best performance for both high- and low-abundance microbes. In addition, developers may analyze in depth the misassemblies and errors in assembled contigs with the ground truth provided by Meta-NanoSim to improve their algorithms. The effect of chimeric reads, as a common source of misassemblies, can be easily evaluated with simulated reads.

## Conclusions

Meta-NanoSim is an ONT metagenomic simulator that simulates complex microbial communities with read features true to the platform. Given a training dataset, Meta-NanoSim generates read length distributions, error profiles, and alignment ratio models by default. Optionally, it also detects chimeric reads and quantifies species abundance levels. Meta-NanoSim aims to capture platform-specific features and can be adopted to profile datasets from any ONT sequencing chemistry and basecallers tested to date. Considering the evolving Nanopore sequencing technology, with base accuracy improvements afforded by newer flowcells and updated chemistries, it is imperative to factor in those changes when simulating data with characteristics that are as close as possible to experimental data. The NanoSim suite of tools has this ability, which is accomplished by retraining new models on the latest available sequencing data. Pretrained models are available and will be supported along with future NanoSim releases to account for nanopore technology advancements. The performance of metagenomic quantification of Meta-NanoSim surpasses the performance of the current state of the art. Meta-NanoSim is the first ONT metagenomic read simulator that can simulate chimeric reads and abundance levels at base level. Chimeric read detection improves the read length modeling and helps reproduce such features in simulated reads to challenge metagenomic assemblers, taxonomy binners, and abundance quantification tools. The tool also supports multiprocessing and streamed reference genomes from online servers to speed up simulations when hundreds or thousands of genomes are to be simulated in a microbial community. By comparing simulated reads with empirical datasets, we show that Meta-NanoSim preserves some key characteristics of ONT metagenomic reads well. Further, our metagenomic assembly benchmarks demonstrate a use case and utility of Meta-NanoSim. We expect Meta-NanoSim to have broad utility in the development, testing, and improvement of such applications.

## Data Availability and Requirements

Project name: Meta-NanoSim

Project homepage: https://github.com/bcgsc/NanoSim

Operating system: Platform independent

Programming language: Python

Other requirements: https://github.com/bcgsc/NanoSim/blob/master/README.md

**License**: GNU General Public License

SciCrunch RRID:SCR_018243

BioToolsID: meta-nanosim

The project is accessible through the Code Ocean capsule [35].

## Abbreviations

bp: base pair; EM: expectation-maximization; GB: gigabytes; GPU: graphics processing unit; M: million; NGA50: length of the shortest alignment block for which longer or equal-length alignment blocks cover 50% of the reference genome size; nt: nucleotides; ONT: Oxford Nanopore Technologies; TB: terabytes.

## Supplementary Material

giad013_GIGA-D-22-00275_Original_Submission

giad013_GIGA-D-22-00275_Revision_1

giad013_Response_to_Reviewer_Comments_Original_Submission

giad013_Reviewer_1_Report_Original_Submission Andre Rodrigues-Soares -- 11/26/2022 Reviewed

giad013_Reviewer_2_Report_Original_Submission Hadrien GourlÃ -- 12/5/2022 Reviewed

giad013_Supplemental_File

## Additional Files

Meta-NanoSim is implemented in Python within the NanoSim suite. The source code and pretrained models used in this study are available on GitHub: https://github.com/bcgsc/NanoSim. An archival copy of the code is also available via the *GigaScience* database GigaDB [36]. NanoSim version 3.0.2 is used for this work. Meta-NanoSim is platform independent and is released under the GNU GPL license. The data analyzed during this study are described in the manuscript and supplementary methods in Additional File 1.

**Supplementary Table S1**. Statistical analysis of the abundance estimation on the *Adp* dataset.

**Supplementary Table S2**. MetaFlye assembly comparison.

**Supplementary Figure S1**. Chimeric read detection and circularity check.

**Supplementary Figure S2**. Average length distribution for each species.

**Supplementary Figure S3**. Abundance estimation comparison.

**Supplementary Figure S4**. Performance of Meta-NanoSim in simulating 1 million reads.

**Supplementary Figure S5**. Quality score distribution for 1 million Meta-NanoSim simulated reads.
