# Supplementary material for: Characterization and simulation of metagenomic nanopore sequencing data with Meta-NanoSim
Source: Gigascience. 2023 Mar 20;12:giad013. doi: 10.1093/gigascience/giad013 (PMC10025935; doi:10.1093/gigascience/giad013)
Supplement: giad013_GIGA-D-22-00275_Revision_1 [file giad013_giga-d-22-00275_revision_1.pdf]

GigaScience

Characterization and simulation of metagenomic nanopore sequencing data with Meta-NanoSim

--Manuscript Draft--

**Manuscript Number:**	GIGA-D-22-00275R1
**Full Title:**	Characterization and simulation of metagenomic nanopore sequencing data with Meta-NanoSim
**Article Type:**	Technical Note
**Funding Information:**	Genome Canada (281ANV) — Dr. Inanc Birol<br>Genome British Columbia (281ANV) — Dr. Inanc Birol<br>National Human Genome Research Institute (R01HG007182) — Dr. Inanc Birol
**Abstract:**	Background<br><br>Nanopore sequencing is crucial to metagenomic studies as its kilobase-long reads can contribute to resolving genomic structural differences among microbes. However, sequencing platform-specific challenges, including high base-call error rate, non-uniform read lengths, and the presence of chimeric artifacts, necessitate specifically designed analytical algorithms. The use of simulated datasets with characteristics that are true to the sequencing platform under evaluation is a cost-effective way to assess the performance of bioinformatics tools with the ground truth in a controlled environment.<br><br>Results<br><br>Here, we present Meta-NanoSim, a fast and versatile utility that characterizes and simulates the unique properties of nanopore metagenomic reads. It improves upon state-of-the-art methods on microbial abundance estimation through a base-level quantification algorithm. Meta-NanoSim can simulate complex microbial communities composed of both linear and circular genomes and can stream reference genomes from online servers directly. Simulated datasets showed high congruence with experimental data in terms of read length, error profiles, and abundance levels. We demonstrate that Meta-NanoSim simulated data can facilitate the development of metagenomic algorithms and guide experimental design through a metagenome assembly benchmarking task.<br><br>Conclusions<br><br>The Meta-NanoSim characterization module investigates read features including chimeric information and abundance levels, while the simulation module simulates large and complex multi-sample microbial communities with different abundance profiles. All trained models and the software are freely accessible at Github: https://github.com/bcgsc/NanoSim.
**Corresponding Author:**	Saber Hafezqorani<br>Canada's Michael Smith Genome Sciences Centre<br>Vancouver, BC CANADA
**Corresponding Author Secondary Information:**	
**Corresponding Author's Institution:**	Canada's Michael Smith Genome Sciences Centre
**Corresponding Author's Secondary Institution:**	
**First Author:**	Chen Yang
**First Author Secondary Information:**	

Powered by Editorial Manager® and ProduXion Manager® from Aries Systems Corporation

**Order of Authors:**	Chen Yang
	Theodora Lo
	Ka Ming Nip
	Saber Hafezqorani
	René L Warren
	Inanc Birol
**Order of Authors Secondary Information:**	
**Response to Reviewers:**	Dear Dr. Zauner,<br><br>We would like to thank you for your consideration of our manuscript. The thorough and constructive feedback provided by you and the reviewers is greatly appreciated. In our revised submission, we have addressed the concerns raised, as outlined below in this letter, and have made the necessary revisions to the manuscript. Please note that the edited portions of the manuscript are highlighted in red, to make it easier to identify the changes compared to the original submission. In addition, our responses to the comments contain references to the line numbers of the changes made.<br><br>We would like to inform you that in accordance with your suggestion, we have included the CodeOcean capsule in the "availability" section and the DOI link in the bibliography, citing the capsule as per your suggestion. Additionally, we have deleted the sentence "Any restrictions to use by non-academics: Please contact the authors," from the availability section, since we use the GNU public license.<br><br>With regard to software registration in scientific resources, we would like to inform you that Meta-NanoSim has already been registered at the SciCrunch database and the RRID information has been included along with the FTP submission. All three NanoSim tools (NanoSim, Trans-NanoSim, and Meta-NanoSim) have also been registered at the bio.tools database with their corresponding identifiers. We have included this information in the "availability" section of the manuscript. Lastly, we would like to note that, as pertains to the workflowhub.eu, the Meta-NanoSim (and NanoSim) code has been implemented as a suite of modules, and there is no workflow being introduced in our manuscript.<br><br>Sincerely,<br><br>Saber Hafezqorani, Chen Yang, and Inanc Birol<br>Canada’s Michael Smith Genome Sciences Centre<br>British Columbia Cancer Agency<br><br>REVIEWER 1<br><br>•On line 64. Given the range of samples that can currently be sequenced using Nanopore sequencing and the recent focus on short reads as opposed to the previous highlight given to long reads, this statement is out-of-date. I would recommend describing instead the current range of sizes that can be sequenced via Nanopore.<br><br>Response: We clarified in the main text that ONT reads can have variable lengths. We also expanded on the N50 length metric definition. (Lines 63-65)<br><br>•How the authors would approach the different error rates namely of different types of flowcell (R9 vs R10). While I don't think this should be central to the development of the tool, I think this should be addressed in the manuscript in some way.<br><br>Response: Thank you for your comment about the error rates of different flowcells, which is an important point to consider as the nanopore technology matures. Indeed, Nanopore sequencing is an evolving technology and the error rates have improved tremendously since its emergence. Therefore, we agree that a simulator should be flexible to future advancements in this technology.<br><br>NanoSim is designed specifically to address this concern by learning and

Powered by Editorial Manager® and ProduXion Manager® from Aries Systems Corporation

characterizing features of the input training data. Given a set of nanopore reads from a new flowcell (e.g. R10), the characterization stage in NanoSim (the “read_analysis.py” script) can learn the features that are native to the platform. If users have any specific flowcell chemistry that they want to simulate, they can use the characterization module to train their own models for simulation.

For the convenience of our users, we have been providing ready-to-use pre-trained models for the past 5 years. We will continue to provide pre-trained models for more recent flowcells and respective chemistries in future releases of NanoSim.

We had initially addressed this concern when explaining the NanoSim workflow (Implementation section, Meta-NanoSim general design sub-section and Fig. 1) as well as in the Conclusion section. We have further expanded on this important point and added the following sentence for clarification (Lines 558-564):

“Considering the evolving Nanopore sequencing technology, with base accuracy improvements afforded by newer flowcells and updated chemistries, it is imperative to factor in those changes when simulating data with characteristics that are as close as possible to experimental data. The NanoSim suite of tools has this ability, which is accomplished by re-training new models on the latest available sequencing data. Pre-trained models are available, and will be supported along with future NanoSim releases to account for nanopore technology advancements.”

•I would also have liked to see distributions of PHRED quality scores in the simulated reads in the analyses conducted in the manuscript.

Response: The simulated reads for our metagenome assembly benchmarking were generated in FASTA format. Therefore, quality scores do not contribute to the assembly performance of meta-Flye shown in Figure 5. We have investigated the distribution of quality scores in 1 million simulated reads based on the trained model for the Log dataset (Supplementary Figure S5). Overall, aligned reads tend to have higher quality scores than their unaligned counterparts. This is expected because a higher phred quality score corresponds to a lower likelihood of sequencing base errors (Lines 429-431).

REVIEWER 2:

•Line 166-168: the clause about structural variant is unclear to me, and perhaps to the reader. Please consider rephrasing.

Response: Thanks for your comment. We added clarity to the sentence and rephrased it as follows (Lines 167-171):

“They may arise because of sequencing artifacts, or may appear like structural variants when the source genome is absent from the reference metagenome. More specifically, sequencing reads may be mapped to a similar but structurally different genome (e.g. a different strain or subspecies), causing segments of the read to align to different regions of the reference genome.”

•Line 209-210: I understand that the number of mapped reads is unadequate for abundance estimation of ONT data, but k-mers should not suffer from the same problem, shouldn't they? the number of k-mers matching to a genomic region (or a genome) will scale appropriately with read length. I have therefore a hard time understanding why k-mers are presented as problematic in the first sentence of the Abundance Estimation paragraph.

Response: There are two main reasons for this. First, as mentioned in the first sentence of that paragraph, the existing abundance estimation methods generally quantify the number of k-mers under the presumption that all reads have equal lengths, which is not a feature of ONT reads. Therefore, these algorithms originally designed for short reads are not directly applicable to ONT reads. Second, erroneous bases (due to mismatch or indel errors) in ONT reads would contribute to many low-multiplicity k-mers that do not belong to the reference. In other words, the k-mer multiplicities in long

reads will very likely be an underestimate of the underlying microbial abundance levels. However, we acknowledge that this issue may be alleviated with improvements in the technology when the reads have much lower error rates.

•Line 379: What do you mean by "pronounced". Please consider rephrasing.

Response: Thanks for your comment, we rephrased the sentence as follows (Lines 380-383):

“The distribution of abundance deviations for experimental data and Meta-NanoSim simulated reads are statistically the same (Kolmogorov-Smirnov test p-value = 0.787), while the distributions for CAMISIM-simulated reads and experimental data are noticeably inconsistent, as CAMISIM does not provide this feature.”

•Line 438: geomes → genomes

Response: Thank you. We corrected the spelling. (Line number: 445)
We also corrected a couple of other spellings and added some punctuation marks. (Lines: 131, 166, 181, 217, 288, and 454)

•Figure 2: panel 2 should have the same theme as the other subplots of the figure

Response: We updated the subplot to account for this.

Software comments:

•I'd like to be able the examples present in the documentation out of the box: please add a link and instructions on how to download and unpack the zymo community

Response: The Supplemental Methods section in Additional File 1 describes the data analysed in this study. Thanks to your comment, we also added more information including instructions on how to download and access the Zymo mock community in the above-mentioned section.

•Speaking of the zymo community, why do Campylobacter and S. cerevisiae have a different path than the other genomes in the examples?

Response: The Zymo mock community does not contain Campylobacter spp. We assume you are referring to Cryptococcus neoformans. The reference genomes for Cryptococcus neoformans and S. cerevisiae provided by Zymo community are draft assemblies. We noticed that they are very fragmented and thus they cannot be used for quantification. Therefore, we downloaded their reference genomes from RefSeq, which resulted in the different paths. We clarified this in the Supplemental Methods - Datasets section (Additional File 1).

•If you plan to not update the pre-trained error models to a more recent version of scipy, please pin scipy 0.22.1 to the bioconda recipe, so that users can use pre-trained models out-of-the-box

Response: Thanks for flagging this technical detail. We assume you are referring to the scikit-learn package incompatibility issue but not scipy. In either case, please kindly note that we will include newly pre-trained models along with the next NanoSim release, which uses the latest versions of the required packages.

In the meantime, we also updated the NanoSim Github page with comprehensive information on how to avoid package incompatibility issues. We also updated the “requirements.txt” file used for installing NanoSim and relaxed unnecessary package version requirements, which should address this issue.

•Please make a new release of the software including pull request !67

Response: Thanks for pointing this out. Please note that pull request 167 is already solved and merged into the master branch. This will be automatically included in the

	next NanoSim release.<br><br>•In a future version of Nanosim, I urge you to consider gathering all scripts into subcommands (i.e. nanosim simulate [--params] instead of simulate.py [--params]. I realise this a big breaking change but it is good practise, and avoids polluting a user's PATH with many scripts. This change is in my opinion not required for the paper to be published, but something I'd like you to consider for a future release.<br><br>Response: Thanks for this great advice and suggestion. We will incorporate this in the next NanoSim release.
**Additional Information:**	
**Question**	**Response**
Are you submitting this manuscript to a special series or article collection?	No
**Experimental design and statistics**<br><br>Full details of the experimental design and statistical methods used should be given in the Methods section, as detailed in our Minimum Standards Reporting Checklist. Information essential to interpreting the data presented should be made available in the figure legends.<br><br>Have you included all the information requested in your manuscript?	Yes
**Resources**<br><br>A description of all resources used, including antibodies, cell lines, animals and software tools, with enough information to allow them to be uniquely identified, should be included in the Methods section. Authors are strongly encouraged to cite Research Resource Identifiers (RRIDs) for antibodies, model organisms and tools, where possible.<br><br>Have you included the information requested as detailed in our Minimum Standards Reporting Checklist?	Yes
**Availability of data and materials**<br><br>All datasets and code on which the	Yes

conclusions of the paper rely must be either included in your submission or deposited in publicly available repositories (where available and ethically appropriate), referencing such data using a unique identifier in the references and in the “Availability of Data and Materials” section of your manuscript.<br><br>Have you have met the above requirement as detailed in our Minimum Standards Reporting Checklist?	

Powered by Editorial Manager® and ProduXion Manager® from Aries Systems Corporation

Manuscript
Click here to access/download;Manuscript;meta-nanosim_for_publication.docx

Characterization and simulation of metagenomic nanopore sequencing data with Meta-NanoSim

Chen Yang[1,2], Theodora Lo[1,2], Ka Ming Nip[1,2], Saber Hafezqorani[1,2], René L Warren[1], Inanc Birol[1,3*]

1. 570 W 7th Ave, Canada's Michael Smith Genome Sciences Centre, BC Cancer, V5Z 4S6, Vancouver, BC, Canada

2. Bioinformatics Graduate Program, University of British Columbia, Vancouver, BC, Canada

3. Department of Medical Genetics, University of British Columbia, Vancouver, BC, Canada

* Corresponding author

Chen Yang: cheny@bcgsc.ca

Theodora Lo: tlo@bcgsc.ca

Ka Ming Nip: kmnip@bcgsc.ca

Saber Hafezqorani: shafezqorani@bcgsc.ca

René L Warren: rwarren@bcgsc.ca

Inanc Birol: ibirol@bcgsc.ca

ORCID iDs: Chen Yang [0000-0002-5144-7748]; Theodora Lo [0000-0002-0534-9557]; Ka Ming Nip [0000-0002-1574-3363]; Saber Hafezqorani [0000-0003-0553-9227]; René L Warren [0000-0002-9890-2293]; Inanc Birol [0000-0003-0950-7839];

ABSTRACT

Background: Nanopore sequencing is crucial to metagenomic studies as its kilobase-long reads can contribute to resolving genomic structural differences among microbes. However, sequencing platform-specific challenges, including high base-call error rate, non-uniform read lengths, and the presence of chimeric artifacts, necessitate specifically designed analytical algorithms. The use of simulated datasets with characteristics that are true to the sequencing platform under evaluation is a cost-effective way to assess the performance of bioinformatics tools with the ground truth in a controlled environment.

Results: Here, we present Meta-NanoSim, a fast and versatile utility that characterizes and simulates the unique properties of nanopore metagenomic reads. It improves upon state-of-the-art methods on microbial abundance estimation through a base-level quantification algorithm. Meta-NanoSim can simulate complex microbial communities composed of both linear and circular genomes and can stream reference genomes from online servers directly. Simulated datasets showed high congruence with experimental data in terms of read length, error profiles, and abundance levels. We demonstrate that Meta-NanoSim simulated data can facilitate the development of metagenomic algorithms and guide experimental design through a metagenome assembly benchmarking task.

Conclusions: The Meta-NanoSim characterization module investigates read features including chimeric information and abundance levels, while the simulation module simulates large and complex multi-sample microbial communities with different abundance profiles. All trained models and the software are freely accessible at Github: https://github.com/bcgsc/NanoSim.

KEYWORDS

Metagenomics, Oxford nanopore sequencing, microbial abundance estimation, sequence simulation, chimeric reads

BACKGROUND

Empowered by the rapid development of next-generation sequencing technologies, metagenomic analysis has enabled comprehensive investigation of the genetic composition and abundance of microbial communities. Metagenomic sequencing bypasses the need to culture each individual species by extracting DNA directly from their natural habitat, making it feasible to study microbes that cannot be isolated or cultured in the laboratory [1,2]. Within the past few decades, the improved throughput and reduced cost of next-generation DNA / RNA sequencing platforms has enabled a wide range of metagenomic studies of environmental, pharmaceutical, and medical relevance [3–5].

Until recently, Illumina short-read sequencing (Illumina Inc., San Diego, CA) has been the technology of choice for metagenomic sequencing projects due to its high throughput, low cost, and low error rate. However, the reads generated by Illumina instruments are often too short (<250 bp) to span inter- and intra-chromosomal homologous regions and suffer from intrinsic biases, thus complicating downstream assembly and taxonomic analysis [6]. As a third-generation long-read sequencing technology, nanopore sequencing from Oxford Nanopore Technologies Ltd. (ONT, Oxford, UK), is gaining traction in metagenomic research efforts, due largely to the long read lengths it generates, as well as the portability of some of their sequencing

platforms [7]. ONT reads have highly variable lengths, ranging from tens to millions of base-pairs (bp). For example, the N50 length (where 50% of bases sequenced are from reads of this length or longer) of ONT reads in a typical run is over 5 kbp [8] and the reported maximum read length exceeds 2 Mbp. At the high end, whole bacterial or viral genomes may be captured by a few sequencing reads [9,10], making it possible to disambiguate between closely-related strains. Since its introduction, ONT sequencing has played an essential role in real-time pathogen identification and clinical diagnosis, including research efforts during the COVID-19 pandemic [11–14].

Although a plethora of metagenomic analysis tools have been developed for short-read sequencing data, the challenges associated with ONT reads, such as high error rate, non-uniform error distributions, and chimeric read artifacts [8,15–17], call for analytical tools designed specifically for long reads. For example, quantification of microbial abundance levels, or metagenomic abundance estimation, is traditionally computed by counting the number of mapped reads followed by fine-tuning of ambiguous mappings [18,19]. This approach has been proven to be cost-effective for Illumina short reads because of their uniform lengths. However, the accuracy of these tools would be understandably impacted when applied on ONT reads, especially for lowly represented genomes, because of the variable lengths and relatively high error rates (5 - 15% depending on the flowcell chemistry and basecalling algorithm) compared to that of Illumina reads (typically less than 1%). In addition, ONT sequencing projects on genomes, transcriptomes, and metagenomes, from prokaryotes to eukaryotes, were all reported to contain certain problematic reads with gapped or chimeric alignments, likely generated due to library

preparation or sequencing artifacts [17,20–25]. Reference-based abundance estimation using merely primary alignments may further be affected by the presence of these chimeric reads, as well as reads that span the start position of a circular genome. To the best of our knowledge, even the state-of-the-art program, MetaMaps, does not account for chimeric reads, but simply uses an Expectation-Maximization (EM) algorithm to disambiguate multi-mapped reads [26]. In this work, we show that there is still room for improving metagenomic abundance estimation, a proposition attainable by quantifying aligned bases instead of reads, while leveraging chimeric read information.

In the process of tool development and benchmarking, a metagenomic ONT read simulator and associated simulated datasets with known ground truth can save time and money. Ideally, such a read simulator should reflect the true characteristics of the ONT platform and allow effective evaluation of bioinformatics tools. In return, the evaluation results can guide the experimental design of metagenomics projects, to help determine the desired sequencing depths and number of replicates [27].

Currently, the only simulator that specifically simulates ONT metagenomic datasets is CAMISIM [28]. The workflow of CAMISIM is focused on the composition design of a microbial community given a taxonomy profile, while the abundance levels are drawn from a lognormal distribution. The obvious drawback of this approach is that users cannot request the abundance levels as they need. CAMISIM uses NanoSim [15] as its engine to simulate ONT reads for each genome separately once the composition of the community is determined. Following the same idea, one

can also use other existing ONT genomic simulators naively to simulate each composite genome separately and then aggregate the reads according to the desired abundance. However, it is impractical to simulate a large microbial community with hundreds or more genomes with this approach, not to mention that the existing simulators for ONT reads are not designed to model metagenomic specific features, such as chimeric reads and deviations in abundance levels. More importantly, the simulation of abundance levels should be consistent with the quantification method, thus merely mixing the reads from different genomes will yield a compromised abundance profile. Taken together, we note that the previous version of NanoSim can be upgraded to capture and simulate read properties specific to metagenomics, especially the microbial abundance levels and chimeric reads – two key factors that may influence metagenome assembly, taxonomy binning, and abundance estimation. Further, in real world scenarios, viruses, bacteria, and fungi co-exist in complex microbial communities, hence the ability to simulate complex metagenomes comprising both circular and linear genomes is very important.

Here, we introduce Meta-NanoSim (released within NanoSim version 3), an ONT metagenome simulator for complex microbial communities. Given a training dataset, Meta-NanoSim characterizes read length distributions, error profiles, and alignment ratio models. Optionally, it also detects chimeric reads and estimates microbial abundance levels. In our benchmarks, the performance of the metagenomic abundance estimation feature of Meta-NanoSim surpasses the current state-of-the-art methods. The chimeric read detection feature also improves the read length modelling, and thus simulating this artifact of the technology may challenge metagenomic analytical tools with a real-world scenario. Through benchmarking experiments comparing

simulated reads with empirical datasets, we show that Meta-NanoSim preserves the key characteristics of ONT metagenomic reads. Finally, we showcase the usability and utility of Meta-NanoSim in assessing the performance of a metagenomic assembly tool.

IMPLEMENTATION

Meta-NanoSim general design

Meta-NanoSim is implemented in Python as the "`meta`" sub-module for both characterization and simulation stages within the NanoSim suite. It learns the technical and metagenomic-specific features of ONT reads in the characterization stage, builds statistical models, and applies them in the simulation stage (**Fig. 1**). In the characterization stage, it takes ONT metagenomic reads and a reference metagenome as input to infer the ground truth through sequence alignments. Based on those alignments, the read length distributions (for aligned and unaligned reads) and basecall events are modelled via kernel density estimation and mixture statistical models, respectively. In addition to existing NanoSim features, we introduce two new analyses in the characterization stage: chimeric read analysis for genome/metagenomes and abundance estimation for metagenomic datasets.

Simulating a metagenomic dataset with Meta-NanoSim requires four inputs: (i) a list of reference genomes to be simulated (local file paths or web addresses), (ii) target abundance levels, (iii) genome topologies (i.e., linear or circular), and (iv) the pre-trained model from the characterization stage. Meta-NanoSim can optionally stream reference genome sequences from

either RefSeq [29] or Ensembl [30] automatically without requiring extra disk storage, which facilitates large microbial community simulations. Since microbial sequencing projects are often carried out in a multi-sample or multi-replicate fashion, Meta-Nanosim is designed to simulate multiple samples in one batch with user-defined abundance level profiles as input.

Fig. 1 Meta-NanoSim workflow. Meta-NanoSim consists of two stages: characterization and simulation. In the characterization stage, given a training dataset and reference metagenome, Meta-NanoSim builds models for the read length distributions and basecall events. It optionally profiles chimeric read artifacts and quantifies an abundance profile. It can also calculate the deviation between expected and estimated abundance levels. In the simulation stage, Meta-NanoSim takes four inputs: (i) a list of genomes to be simulated, (ii) a list of genome topologies, (iii) target abundance profiles, and (iv) the models generated from the characterization stage. Meta-NanoSim outputs simulated reads and error profiles.

Chimeric read detection and simulation

A chimeric read, also known as a "split read", has two or more sub-alignments against distinct regions of the reference genome/metagenome. They may arise because of sequencing artifacts, or may appear like structural variants when the source genome is absent from the reference metagenome. More specifically, sequencing reads may be mapped to a similar but structurally different genome (e.g. a different strain or subspecies), causing segments of the read to align to different regions of the reference genome. When the query and reference coordinates of sub-alignments do not overlap, we define them as a set of compatible alignments. Finding the best

compatible alignment set problem is akin to the interval scheduling maximization problem, which finds a set of non-overlapping intervals of maximum size. For each read, we exhaustively search for all compatible alignments for each sub-alignment to generate a list of compatible alignment sets (**Fig. S1** in Additional File 1). We then select the best element from the list for downstream analysis, based on alignment quality and total aligned length. If, for a given read, the best element contains two or more compatible alignments, the read is considered as chimeric and its aligned length, gap length, and source species (specific to metagenome mode) are modelled for simulation. Exceptions are reads bridging the start and end of a circular genomic reference; these reads are detected but not designated as chimeric; the sub-alignments within these reads are concatenated as single alignments.

To determine the source species for each segment in chimeric reads, we built a simplified hidden Markov model where the start probability is the input abundance, the emission probability represents which species the next segment is coming from given the previous one, and the transitional probability of species is the change of abundance in the underlying Markov chain. Since the species to be simulated, namely the states in a Markov model, may be different between the training and simulation metagenome, we generalize the emission probability as a single value called shrinkage rate s ($0 < s <= 1$). This parameter describes the reduction of abundances (probabilities) of other species, while maintaining the relative abundances among them. Assuming the input abundance is $\{p_A, p_B, p_C, \ldots, p_N\}$ for n species, when the first segment comes from species A, the transitional probabilities for the other species would become $\{s\times p_B, s\times p_C \ldots s\times p_N\}$ and the transitional probability for A would be inflated as $1 - s\times \sum_{i=B}^{N} p_i$. To learn

s, all segments in chimeric reads are divided into overlapping pairs, and the probability for the source species of the second segment being different from the first one is recorded. In this way, we can calculate the reduction of abundance for every species. The average reduction is the shrinkage rate, and the inflated abundance for being from the same species can be inferred as well. The shrinkage rate can also be adjusted by the user, to 1 for example, if one assumes all DNA molecules are homogeneously suspended in the buffer.

In summary, Meta-NanoSim first determines the number of segments to be simulated based on a geometric distribution. A read is chimeric if it has two or more segments. Then, Meta-NanoSim generates the lengths of each segment and the gap(s) between them using kernel density estimation learnt from empirical reads. The source species of the first segment is randomly picked based on the input abundance level. Starting from the second segment, the abundance levels are re-computed based on the previous species and *s*. The source species is determined one after another, and then sequences are extracted, mutated with purposely introduced errors, and collated in the same process as non-chimeric reads.

Abundance estimation

Existing abundance estimation methods generally quantify the number of mapped reads or *k*-mers, under the presumption that all reads have equal lengths. However, since the ONT read length varies across several orders of magnitude, the mean read length for each species is likely to be different. When all species are equally and deeply sequenced, according to the central limit theorem, the standard deviation of mean lengths would scale with $1/\sqrt{n}$, where n is the number

of species. For finite data, low-abundance species may have a higher standard deviation because there are fewer sequences representing them. We observe the mean read lengths of uniquely aligned reads to vary substantially in datasets where species abundance levels are logarithmically distributed, necessitating base-level instead of read-level quantification algorithms (**Fig. S2** in Additional File 1).

Another key challenge that confounds short-read metagenomic analysis is ambiguously aligned reads. In ONT datasets, however, most reads are long enough to span inter- and intra-species homologous regions, and the chimeric read detection feature can resolve the estimation for reads having multiple sub-alignments and for reads bridging the start and end of a circular genome. For the remaining small fraction of multi-aligned reads between closely-related species, the estimation for them can be optimized using the EM algorithm.

The EM algorithm (**Algorithm 1**) first processes uniquely aligned reads to calculate a baseline abundance profile. Then it starts the expectation step, which is to assign multi-aligned bases proportionally to their respective species based on their relative abundances. Next, in the maximization step, these multi-aligned bases are used to update the abundance profile. The algorithm then goes back to the expectation step to update the fractions of multi-aligned bases based on the new abundance profile. The E and M steps alternate until the difference in abundances between two rounds is lower than a threshold (default: 1%). Note that the abundance levels are in the units of relative genomic DNA weight, and they can be used to calculate genome copy numbers when divided by the respective genome sizes.

Algorithm 1 EM for metagenome abundance estimation

```
abundance_list = {species1: abundance1; species2: abundance2; …}
base_count = {species1: count1; species2: count2; …}

Start processing uniquely aligned reads:
   for each uniquely aligned read and its source species:
      base_count[species] += aligned bases
   abundance_list = {species: base_count[species]/ sum(base_count[species])}

Start processing multi-aligned reads
while diff >= min(abundance_list.values()) * 0.01:
   E-Step:
      for each multi-aligned read:
         read_abun = the sum of abundances for all possible species for that read
         for each possible species:
            fraction = aligned bases * abundance_list[species] / read_abun
            base_count[species] += fraction
   M-Step:
         abundance_list = {species: base_count[species]/ sum(base_count[species])}
         diff = |abundance_list – prev_abundance_list|
```

Meta-NanoSim offers adundance estimation with or without chimeric read detection. When chimeric read detection is enabled, all subalignments are used for computing estimates; otherwise, only primary alignments are used. Meta-NanoSim records the aligned bases for each sub-alignment towards their source genome, and then uses EM algorithm to assign multi-aligned segments proportionally to their putative source genomes iteratively.

Abundance deviation simulation

Meta-NanoSim simulates abundance deviation with user-defined lower and upper deviation boundaries. We noticed a weak positive correlation between genome size and abundance deviation in our analysis. During simulation we first randomly draw a list of relative error between the deviation boundaries. We then assign these errors to each genome based on their sizes,

namely larger deviations are assigned to larger genomes and smaller ones are assigned to smaller genomes. Finally, abundance values are normalized for a total abundance of 100%.

RESULTS

We first assess the performance of the two key features in Meta-NanoSim, chimeric read detection and abundance estimation. To evaluate the similarity between simulated reads and experimental reads, we generated two simulated datasets using models learned from experimental data, and we compared the performance of Meta-Nanosim with that of CAMISIM. We illustrate that Meta-NanoSim is capable of simulating a large complex microbial community containing 125 species based on a human saliva sample. Finally, we showcase an application of Meta-NanoSim simulated data in benchmarking the long-read metagenomic assembler MetaFlye [31]. Specifically, we evaluated the assembly quality and scalability of MetaFlye with respect to increasing sequencing depths.

Evaluation of chimeric read characterization and simulation

Previous studies reported that chimeric reads represent a non-negligible fraction of ONT sequencing datasets ranging from 1.70% to 8.17% depending on the sequencing kits and identification thresholds [17,20,22,23]. In the metagenome datasets used in our study, after ruling out structural variants, we have identified a similar fraction of reads in this category: 2.17% (75,628 reads) in the *Even* dataset and 1.67% (68,444 reads) in the *Log* datasets. These reads are free of known adapters, so their presence may impact downstream analyses, such as assembly, taxonomy binning, and quantification, even after adapter trimming. When aligned to their

respective reference genome sequence(s), ONT reads may contain unaligned or soft-clipped regions. In our tests, the chimeric read detection feature of Meta-NanoSim significantly reduced the length of these unaligned regions, which explained why some of the reads have over 1 kbp long unaligned portions (**Fig. 2A**). As seen in **Fig. 2B**, the length distributions of the gaps between split alignments follow multi-modal distributions. Meta-NanoSim uses kernel density estimation to model them, with results exhibiting strong similarity between the length distributions of simulated and experimental sequences. We also noticed that the number of segments each read contains can be described as a geometric distribution and the mean probability can be learnt from experimental data (**Fig. 2C**). On average, each read contains 1.03 segments for both data sets under study.

Based on the source species to which each split alignment belongs, chimeric reads can be classified as "intra-species-chimeric" or "inter-species-chimeric". It is observed that the source species of the first segment is affected by the abundance level, while subsequent segment is more likely to be influenced by the identity of the previous species (**Fig. 2D**). We postulate that this is because DNA molecules of the same species are more likely to gather near the nanopore than being homogeneously dispersed in the buffer. Regardless of the actual cause, this phenomenon can be approximated as a simplified hidden Markov model with a generalized emission probability, which is defined as shrinkage rate s here. To our calculation, s is equal to 0.77 for the *Even* dataset and 0.73 for the *Log* dataset, suggesting that its value may be stable across datasets.

Fig. 2 Evaluation of chimeric read detection and simulation. A. The length distribution of the unaligned regions of reads with or without chimeric read detection for the Log dataset (x-axis in logarithmic scale). **B.** The performance of gap length simulation for the Log dataset (x-axis in logarithmic scale). **C.** The number of segments each read contains for the Even and Log datasets. **D.** All segments in chimeric reads in the Even dataset are converted into overlapping pairs. Each facet represents one source species of the first segment and the x-axis represents the source species of the second segment. Each facet shows the probability of the second segment given the source species of the first one. Cryptococcus neoformans and Saccharomyces cerevisiae are excluded here due to their low abundances.

Evaluation of abundance estimation

We compared the performance of four abundance estimation methods: Meta-NanoSim estimation with chimeric read detection, Salmon quantification with the “—meta” option (Salmon) [32], the base-level estimation reported in the paper that released the dataset (denoted as “Data Note” from here on) [8], and MetaMaps. For Meta-NanoSim estimation, we performed an ablation study that removes key components of the algorithm step by step, including estimation on read-level with chimeric read detection (Meta-NanoSim CR) or with EM algorithm (Meta-NanoSim ER), estimation on base-level (Meta-NanoSim B), base-level with chimeric read detection (Meta-NanoSim CB), base-level with EM algorithm (Meta-NanoSim EB), and base-level with chimeric read detection fine-tuned by EM algorithm (Meta-NanoSim ECB). All compared methods, except for MetaMaps, are computed based on Minimap2 alignments. We compared the estimated abundances to the expected values provided by the manufacturer based on R-

squared, standard deviation, and percent error.

In general, all base-level quantification methods (i.e. Meta-NanoSim B, CB, EB, and ECB, and Data Note) performed better than read-level quantification methods (i.e. Salmon, MetaMaps, and Meta-NanoSim CR and ER), and Meta-NanoSim base-level estimations have the highest correlation with the expected abundances (**Table 1**, **Fig. S3** in Additional File 1). For the *Even* dataset, all four Meta-NanoSim base-level methods performed similarly; the stand-alone base-level quantification has the highest R-squared value for the *Even* dataset, while the chimeric read detection helped reduce the percent error, mainly for low-abundance species *Cryptococcus neoformans* (**Fig. S3** in Additional File 1). MetaMaps, as a read-level quantification method designed specifically for ONT metagenomic data, although ranked highest among this category, has twice the percent error than than base-level methods. For the *Log* dataset, Meta-NanoSim base-level estimations also had similar R-squared values, higher than other methods. Although the metrics are very similar for the *Log* dataset, the performance on low-abundance species may be overshadowed by high-abundance species. Therefore, we computed the coefficient of correlation and error between log-transformed estimated and expected abundances. After log-transformation, Salmon, Meta-NanoSim ECB, and Meta-NanoSim EB performed similarly; Salmon has the highest correlation and Meta-NanoSim ECB has the lowest percent error. The metrics for Meta-NanoSim estimation without EM, on the other hand, decreased significantly due to difficulty in differentiating multi-mapped reads for low-abundance species. When estimating the abundance levels for the *Log* dataset, Minimap2 incorrectly assigned 18,212 reads to the *E. faecalis* genome as primary alignments, but these reads can also align to an inter-species

homologous region in the *L. monocytogenes* genome. In fact, *E. faecalis* is a low-abundance species in the *Log* dataset with only 33 unique alignments. Therefore, the methods with EM algorithm resolved the multi-aligned reads problem, indicating that EM can be advantageous for datasets consisting of similar genomes but with large variances in abundance levels.

To recapitulate our findings, we utilized another logarithmically distributed mock microbial community from a previous study [33] (denoted as the *Adp* dataset from here on) and repeated the quantifications with Meta-NanoSim base-level methods, Salmon, and MetaMaps (**Table S1** in Additional File 1). As we had previously observed, all four Meta-NanoSim methods performed similar to each other with the highest correlation to the expected values. MetaMaps quantification, although with the lowest percent error among all compared methods, showed a much lower correlation in terms of R-squared and standard deviation. Taken all together, Meta-NanoSim base-level quantification, after chimeric read detection and fine-tuning by the EM algorithm, had balanced correlation and percent error robustly, thus making it preferrable for naturally occurring microbial communities with varying abundance levels.

Table 1 Statistical analysis of the abundance estimation results compared to expected abundances.

Tool	Algorithm				*Even* dataset			*Log* dataset			
	E	C	B	R	R^2	Std	PE	R^2	Log R^{2*}	Std*	PE
Meta-NanoSim	√	√	√		0.7463	0.0225	144.5317	**1.0000**	0.9920	0.1818	**256.7856**
		√	√		0.7465	0.0225	**144.0877**	0.9999	0.7899	0.9295	53349.95
	√		√		0.7498	**0.0224**	145.6229	**1.0000**	0.9917	0.1843	260.6973
			√		**0.7499**	**0.0224**	145.4656	**1.0000**	0.7895	0.9304	53443.94
		√		√	0.4305	0.0337	326.3432	0.9980	0.7778	0.9560	57527.07
	√			√	0.4396	0.0335	313.1466	0.9980	0.7776	0.9565	57651.72
**Salmon**	√			√	0.4269	0.0339	318.1502	0.9978	**0.9955**	**0.1366**	261.1368
**Data Note**			√		0.6702	0.0257	181.1667	0.9998	0.9863	0.2374	359.9314

**MetaMaps**	√			√	0.4420	0.0334	258.4563	0.9979	0.9652	0.3781	1366.705

R^2: R-squared, **Std**: standard deviation, **PE**: summation of percent error

E: EM algorithm, **B**: base-level quantification, **R**: read-level quantification, **C**: chimeric read detection

* The expected and estimated abundances are log-transformed before calculating R-squared value and standard deviation.

Because of the deviation between expected and estimated abundance levels, we introduce a feature that can simulate this observation. We compared the deviation between expected abundances and experimental data, and the simulation results of NanoSim and CAMISIM (**Fig. 3**). The distribution of abundance deviations for experimental data and Meta-NanoSim simulated reads are statistically the same (Kolmogorov-Smirnov test *p*-value = 0.787), while the distributions for CAMISIM simulated reads and experimental data are noticeably inconsistent, as CAMISIM does not provide this feature.

Fig. 3 Abundance level deviations between experimental and simulated metagenomic reads. In this plot, each dot represents a microbial genome, and the y-axis represents the deviation in percentage between the expected values and experimental/simulated values.

Comparison between simulated and experimental datasets

To demonstrate the performance of Meta-NanoSim, we trained it with the *Log* dataset and compared the simulated datasets against the result of CAMISIM. With eight processors, simulation of one million reads took under 20 minutes (or under 160 CPU-minutes) for Meta-NanoSim, while CAMISIM required more than six hours to complete.

The read lengths of simulated datasets from Meta-NanoSim follow the empirical length

distribution closely, with a median read length peak at 4,040 nt (3,994 nt for empirical reads) (**Fig. 4A**). In contrast, the lengths of CAMISIM-simulated reads deviate far from those of the empirical data. Moreover, the length distribution of unaligned regions on Meta-NanoSim simulated reads captures the patterns in empirical reads well, with multiple peaks below 100 nt. In contrast, the lengths of unaligned part in CAMISIM reads are inflated as it does not detect nor simulate chimeric reads. Both Meta-NanoSim and CAMISIM mimic the mismatch and deletion events well when compared to the empirical dataset (**Fig. 4B**), which demonstrates the robustness of NanoSim mixture statistical models. However, Meta-NanoSim simulates insertion and match events better than CAMISIM, also due to the change of model.

Fig. 4 Performance of Meta-NanoSim and CAMISIM in simulating one million reads from the Log dataset. A. Comparison of read length distributions in the empirical vs. simulated reads (x-axis in logarithmic scale). Unaligned length represents the length of unaligned part of each aligned read. **B.** Cumulative probability distributions of the lengths of matches/errors in empirical and simulated reads.

Additionally, we challenged Meta-NanoSim with two simulation tasks to mimic real-world use cases. First, we simulated two samples at the same time with the pre-trained model from the *Log* dataset. Each sample contained one million reads from the seven species from the *Adp* dataset with different abundance levels. Meta-NanoSim simulation finished within 51 minutes with eight processors. Although the metagenome to be simulated is completely different from the one used for training, simulated reads exhibited similar read features as the experimental data, and the

abundance levels are highly in accordance with the expected values (**Fig. S3** and **Fig. S4** in Additional File 1). Next, we randomly picked a saliva sample from the Human Microbiome Project (HMP) and simulated ONT reads using the same microbial composition [34]. The abundance levels of the 125 different bacteria strains range between 4.28% to 12.49%. By streaming reference genomes from RefSeq directly, it took Meta-NanoSim less than three hours to simulate 10 million reads, including the time used for streaming reference genomes from RefSeq server.

Application in metagenome assembly benchmarking

To demonstrate the utility of Meta-NanoSim, we simulated four sets of data with 1, 2, 4, and 10 million reads (denoted as “1M”, “2M”, “4M”, and “10M” from here on, respectively) based on models learnt from the *Log* dataset to assess the correctness, scalability, and robustness of metaFlye. For the sake of simplicity and uniformity, all simulated reads presented herein were generated in FASTA format. We note that Meta-NanoSim can optionally simulate quality scores for reads in FASTQ format (**Fig. S5** in Additional File 1). With 128 threads, runtimes ranged from one hour to seven hours (**Table S2** in Additional File 1). The maximum resident set size for the 10M dataset was 212 GB, but intermediate files occupied over 10 TB of disk space during consensus-building stage. We also tried to assemble a larger dataset of 20 million reads, however the assembly failed after 30 days with an out-of-memory error on a 1-TB RAM server. According to the log file, the graph simplification was the most time-consuming stage, which lasted for two weeks.

For the 4M dataset, the MetaFlye assembly has a total reconstruction of 27.81 Mbp, which is

equivalent to 43.72% base coverage of the reference metagenome. These metrics are similar to the reported assemblies using the original training dataset with 3.48 million reads (28.20 Mbp assembled length that covered 46.00% of the reference metagenome) [31]. The average fold coverage is positively correlated to the number of sequencing reads and abundance levels, and accordingly, the genome reconstruction fraction and NGA50 length are positively correlated to the average fold coverage (**Fig. 5**). As expected, genomes with less than 1x coverage have very poor reconstructions. Between 1x and 10x coverage, the positive correlation is mirrored in multiple species, including *B. subtilis*, *S. cerevisiae*, *E. coli* and *S. enterica*. When the coverage reaches 10x, metaFlye is able to reconstruct the genome to nearly 100% (*S. cerevisiae* in the 4M dataset, **Fig. 5**). When the coverage reaches 30x, the NGA50 length can cover the whole genome size (*B. subtilis* in the 2M dataset). Similarly, the number of contigs reconstructed for these two species decrease as the number of reads increase, showing how increasing sequencing depth can help assembling genomes into one contig for *B. subtilis* and nearly one contig per chromosome for *S. cerevisiae.* In contrast, when there is insufficient coverage, the assembled genome may be fragmented or even mis-assembled, as in the case of *E. coli* and *S. enterica*. However, a higher coverage does not necessarily lead to a better assembly quality. The reconstruction of *L. monocytogenes* deteriorates with more reads when the fold coverage exceeds 1,000x. Although the genome fraction remains 100%, the NGA50 length is only half or less of the genome size for the 2M, 4M, and 10M datasets. The drop in NGA50 length can be explained by the increasing number of reconstructed contigs and mis-assemblies (**Fig. 5**). With one million reads, only four contigs can be mapped to the *L. monocytogenes* genome, and no mis-assemblies were detected. However, we think that the fold coverage above 1,000X has led to many more misassemblies,

adversely affecting the assembly contiguity as measured by the NGA50 length metric.

Fig. 5 metaFlye assemblies with four sets of simulated metagenome sequencing data. The four sets of simulated datasets include 1, 2, 4 and 10 million reads, respectively. The abundance is the expected abundance level during simulation. The coverage panel shows the average read depth including plasmids (x-axis in logarithmic scale). NGA50 % represents the NGA50 length divided by the reference genome size. Genome fraction is a proportion between the assembled sequences and each corresponding genome. The right-most panel shows the number of misassemblies and assembled contigs as the number of simulated reads increases. BS: Bacillus subtilis, CN: Cryptococcus neoformans, EC: Escherichia coli, EF: Enterococcus faecalis, LF: Lactobacillus fermentum, LM: Listeria monocytogenes, PA: Pseudomonas aeruginosa, SA: Staphylococcus aureus, SC: Saccharomyces cerevisiae, SE: Salmonella enterica.

DISCUSSION

The applications of nanopore sequencing on metagenomic projects are rapidly expanding, motivating the development of metagenomic analysis tools tailored for this specific data type. In this work, we have introduced two main contributions to ONT metagenomic analysis tasks: (i) a new base-level quantification method for metagenomic abundance estimation; and (ii) an upgrade of NanoSim for metagenomic characterization and simulation.

Reference-based metagenomic abundance estimation is key to investigating the microbial composition of an environment, enabled by emerging sequencing technologies. The long read

length of ONT reads provides an opportunity to resolve homologous regions between species or strains, but complications arise due to their high error rates and non-uniform read lengths. Existing methods, primarily developed for short read technologies, generally assume uniform read lengths and therefore only need to count the number of mapped reads or *k*-mers. For example, a 100 bp read and a 10,000 bp read do not have equal contribution to the genome abundance. We have shown that it is necessary to quantify microbial abundances on a base-level rather than read-level to better leverage this data type. In addition to their higher error rates, a small yet substantial fraction of ONT reads are chimeras, which may obscure the accuracy of estimates. The chimeric read detection feature in Meta-NanoSim searches for best compatible alignments and reduces the percent error in microbial abundance estimation. We adopted an EM algorithm to optimize the proportional contributions of ambiguous multi-aligned segments to each potential source species. From our benchmarking results, we demonstrated that the combination of these three components can improve correlations with expected abundances. We note that Meta-NanoSim quantification performs better when the abundance levels are more uniform or when low-abundance microbes do not share large homologous regions with high-abundance microbes. Depending on whether the user wishes to achieve a higher correlation or lower percent error, they can choose to disable or enable chimeric read detection, respectively. Although our work is limited to reference-based quantification, we expect it to inspire the design of reference-free methods and eventually have a broader application.

Built on top of abundance estimation, Meta-NanoSim is able to simulate datasets with desired abundance profiles. It can also recapitulate the abundance level deviation from expected values,

an especially useful feature for designing sequencing projects. When the abundance of a microbial community is known (or estimated), it is essential to determine the sequencing depth that ensures sufficient representation of each species. However, when the sequenced abundance differs from the expected value, simulated data with abundance variations true to the platform can inform the relationship between sequencing depth and abundance levels.

The general workflow of characterization and simulation of Meta-NanoSim follows the same paradigm of the previous versions of NanoSim. The chimeric read detection in characterization stage provides a means to profile all chimeric reads in a library regardless of its root cause. When the reference metagenome is inclusive, the chimeric reads are likely introduced by library preparation and sequencing artifacts; while in reality, since the detection relies on alignment, some chimeric reads may also be attributed to structural variants when the source genome is not present in the reference. In this case, the output of the characterization stage can be used to further investigate such events with specifically designed statistically models and algorithms.

The three new main features added to Meta-NanoSim are (i) chimeric read simulation, (ii) the ability to stream reference genomes from online servers, and (iii) the simulation of a metagenome composed of a mixture of both linear and circular genomes. As chimeric reads may interfere with downstream analyses, simulated datasets with these artifacts are needed for more accurate performance assessment. Characterizing this feature and introducing it to simulated reads will also diversify error types in the reads, helping to improve the robustness of related algorithms. Reference genome streaming is uniquely advantageous when simulating a large

metagenome with hundreds of species. It is a convenient alternative to manual file downloads of reference genomes, and it saves disk space while keeping the runtime reasonable. Similarly, since metagenomes are naturally composed of both linear and circular genomes, having a simulated dataset supporting this important characteristic will add credibility to benchmarking results and better forecast performance with experimental data.

The benchmarking on a metagenome assembly task showcased that Meta-NanoSim can facilitate relevant tool development as well as guide sequencing projects. The resulting assembly quality of Meta-NanoSim simulated reads is comparable to that of the experimental data with similar coverage. Although publicly available mock community sequencing data provide a more realistic training and test set, simulated data provide a ground truth and has virtually no limit in size, making them perfect for testing the accuracy and scalability of algorithms. Through the use of simulated datasets, we demonstrated that metaFlye assembler performs best when the species coverage is between 10x and 1,000x. To ensure a successful assembly of low abundance species, it is suggested to calculate the number of reads needed given an estimated abundance first to ensure just enough coverage without wasting resources. For example, it takes 10 million reads to achieve 10-fold coverage for a 0.1% abundance species with a genome size of 5Mbp. When assembling real microbial communities with highly variable abundance levels, we recommend multiple rounds of assembly with different sample sizes to achieve the best performance for both high- and low-abundance microbes. In addition, developers may analyze in depth the mis-assemblies and errors in assembled contigs with the ground truth provided by Meta-NanoSim to improve their algorithms. The effect of chimeric reads, as a common source of mis-assemblies,

can be easily evaluated with simulated reads.

CONCLUSIONS

Meta-NanoSim is an ONT metagenomic simulator that simulates complex microbial communities with read features true to the platform. Given a training dataset, Meta-NanoSim generates read length distributions, error profiles, and alignment ratio models by default. Optionally, it also detects chimeric reads and quantifies species abundance levels. Meta-NanoSim aims to capture platform-specific features and can be adopted to profile datasets from any ONT sequencing chemistry and basecallers tested to date. Considering the evolving Nanopore sequencing technology, with base accuracy improvements afforded by newer flowcells and updated chemistries, it is imperative to factor in those changes when simulating data with characteristics that are as close as possible to experimental data. The NanoSim suite of tools has this ability, which is accomplished by re-training new models on the latest available sequencing data. Pre-trained models are available, and will be supported along with future NanoSim releases to account for nanopore technology advancements. The performance of metagenomic quantification of Meta-NanoSim surpasses the performance of the current state-of-the-art. Meta-NanoSim is the first ONT metagenomic read simulator that can simulate chimeric reads and abundance levels at base-level. Chimeric read detection improves the read length modelling and helps reproduce such feature in simulated reads to challenge metagenomic assemblers, taxonomy binners, and abundance quantification tools. The tool also supports multiprocessing and streamed reference genomes from online servers to speed up simulations when hundreds or thousands of genomes are to be simulated in a microbial community. By comparing simulated

reads with empirical datasets, we show that Meta-NanoSim preserves some key characteristics of ONT metagenomic reads well. Further, our metagenomic assembly benchmarks demonstrate a use case and utility of Meta-NanoSim. We expect Meta-NanoSim to have broad utility in the development, testing, and improvement of such applications.

AVAILABILITY AND REQUIREMENTS

Project name: Meta-NanoSim

Project home page: https://github.com/bcgsc/NanoSim

Operating systems: Platform independent

Programming language: Python

Other requirements: https://github.com/bcgsc/NanoSim/blob/master/README.md

License: GNU General Public License

SciCrunch RRID: SCR_018243

BioToolsID: meta-nanosim

The project is accessible through the Code Ocean capsule [35].

LIST OF ABBREVIATIONS

bp : basepairs

EM : Expectation-Maximization

GB : gigabytes

GPU : graphics processing unit

M : million

NGA50 : length of the shortest alignment block for which longer or equal length alignment blocks cover 50% of the reference genome size

nt : nucleotides

ONT : Oxford Nanopore Technologies

TB : terabytes

DECLARATIONS

Ethics approval and consent to participate

Not applicable

Consent for publication

Not applicable

Availability of data and material

Meta-NanoSim is implemented in Python within the NanoSim suite. The source code and pre-trained models used in this study are available on Github: https://github.com/bcgsc/NanoSim. An archival copy of the code is also available via the GigaScience database GigaDB [36]. NanoSim version 3.0.2 is used for this work. Meta-NanoSim is platform independent, and is released under the GNU GPL license. The data analysed during this study is described in the manuscript and supplementary methods in Additional File 1.

Competing interests

The authors declare that they have no competing interests.

Funding

This work was supported by Genome Canada and Genome BC [281ANV]; and by the National Human Genome Research Institute of the National Institutes of Health [R01HG007182]. Scholarship funding was provided by the University of British Columbia, and the Natural Sciences and Engineering Research Council of Canada. The content is solely the responsibility of the authors and does not necessarily represent the official views of the funding organizations.

Authors' contributions

IB and CY conceived and designed the study. CY designed and implemented the software with the help of TL, SH, and KMN. KMN and SH provided additional help with the software maintainance. CY drafted the manuscript, and all authors were involved in its revision. All authors read and approved the final manuscript.

Acknowledgements

Not Applicable

REFERENCE

1. Handelsman J. Metagenomics: Application of Genomics to Uncultured Microorganisms. *Microbiol Mol Biol Rev*. 2004; doi: 10.1128/mmbr.68.4.669-685.2004.

2. Chen K, Pachter L. Bioinformatics for whole-genome shotgun sequencing of microbial communities. PLoS Comput. Biol.

3. Schulz F, Alteio L, Goudeau D, Ryan EM, Yu FB, Malmstrom RR, et al.. Hidden diversity of soil giant viruses. *Nat Commun*. 2018; doi: 10.1038/s41467-018-07335-2.

4. Guthrie L, Gupta S, Daily J, Kelly L. Human microbiome signatures of differential colorectal cancer drug metabolism. *npj Biofilms Microbiomes*. 2017; doi: 10.1038/s41522-017-0034-1.

5. Wirbel J, Pyl PT, Kartal E, Zych K, Kashani A, Milanese A, et al.. Meta-analysis of fecal metagenomes reveals global microbial signatures that are specific for colorectal cancer. *Nat Med*. 2019; doi: 10.1038/s41591-019-0406-6.

6. Quince C, Walker AW, Simpson JT, Loman NJ, Segata N. Shotgun metagenomics, from sampling to analysis. Nat. Biotechnol.

7. Brown BL, Watson M, Minot SS, Rivera MC, Franklin RB. MinIONTM nanopore sequencing of environmental metagenomes: A synthetic approach. *Gigascience*. 2017; doi: 10.1093/gigascience/gix007.

8. Nicholls SM, Quick JC, Tang S, Loman NJ. Ultra-deep, long-read nanopore sequencing of mock microbial community standards. *Gigascience*. 2019; doi: 10.1093/gigascience/giz043.

9. Fu S, Wang A, Au KF. A comparative evaluation of hybrid error correction methods for error-prone long reads. *Genome Biol*. 2019; doi: 10.1186/s13059-018-1605-z.

10. Payne A, Holmes N, Rakyan V, Loose M. Bulkvis: A graphical viewer for Oxford nanopore bulk FAST5 files. *Bioinformatics*. 2019; doi: 10.1093/bioinformatics/bty841.

11. Charalampous T, Kay GL, Richardson H, Aydin A, Baldan R, Jeanes C, et al.. Nanopore metagenomics enables rapid clinical diagnosis of bacterial lower respiratory infection. *Nat Biotechnol*. 2019; doi: 10.1038/s41587-019-0156-5.

12. Kafetzopoulou LE, Pullan ST, Lemey P, Suchard MA, Ehichioya DU, Pahlmann M, et al.. Metagenomic sequencing at the epicenter of the Nigeria 2018 Lassa fever outbreak. *Science (80-)*. 2019; doi: 10.1126/science.aau9343.

13. Chan JFW, Yuan S, Kok KH, To KKW, Chu H, Yang J, et al.. A familial cluster of pneumonia associated with the 2019 novel coronavirus indicating person-to-person transmission: a study of a family cluster. *Lancet*. 2020; doi: 10.1016/S0140-6736(20)30154-9.

14. Greninger AL, Naccache SN, Federman S, Yu G, Mbala P, Bres V, et al.. Rapid metagenomic identification of viral pathogens in clinical samples by real-time nanopore sequencing analysis. *Genome Med*. 2015; doi: 10.1186/s13073-015-0220-9.

15. Yang C, Chu J, Warren RL, Birol I. NanoSim: Nanopore sequence read simulator based on statistical characterization. Gigascience.

16. Hafezqorani S, Yang C, Lo T, Nip KM, Warren RL, Birol I. Trans-NanoSim characterizes and simulates nanopore RNA-sequencing data. *Gigascience*. 2020; doi: 10.1093/gigascience/giaa061.

17. Buck D, Weirather JL, de Cesare M, Wang Y, Piazza P, Sebastiano V, et al.. Comprehensive comparison of Pacific Biosciences and Oxford Nanopore Technologies and their applications to transcriptome analysis. *F1000Research*. 2017; doi: 10.12688/f1000research.10571.2.

18. Wood DE, Salzberg SL. Kraken: Ultrafast metagenomic sequence classification using exact alignments. *Genome Biol*. 2014; doi: 10.1186/gb-2014-15-3-r46.

19. Lu J, Breitwieser FP, Thielen P, Salzberg SL. Bracken: Estimating species abundance in metagenomics data. *PeerJ Comput Sci*. 2017; doi: 10.7717/peerj-cs.104.

20. White R, Pellefigues C, Ronchese F, Lamiable O, Eccles D. Investigation of chimeric reads using the MinION. *F1000Research*. 2017; doi: 10.12688/f1000research.11547.1.

21. Martin S, Leggett RM. Alvis: a tool for contig and read ALignment VISualisation and chimera detection. *BMC Bioinformatics*. 2021; doi: 10.1186/s12859-021-04056-0.

22. Marijon P, Chikhi R, Varré JS. Yacrd and fpa: Upstream tools for long-read genome assembly. *Bioinformatics*. 2020; doi: 10.1093/bioinformatics/btaa262.

23. Xu Y, Lewandowski K, Lumley S, Pullan S, Vipond R, Carroll M, et al.. Detection of viral pathogens with multiplex nanopore MinION sequencing: Be careful with cross-Talk. *Front Microbiol*. 2018; doi: 10.3389/fmicb.2018.02225.

24. Tvedte ES, Gasser M, Sparklin BC, Michalski J, Hjelmen CE, Johnston JS, et al.. Comparison of long-read sequencing technologies in interrogating bacteria and fly genomes. *G3 Genes|Genomes|Genetics*. 2021; doi: 10.1093/g3journal/jkab083.

25. Wick RR, Judd LM, Holt KE. Deepbinner: Demultiplexing barcoded Oxford Nanopore reads with deep convolutional neural networks. *PLoS Comput Biol*. 2018; doi: 10.1371/journal.pcbi.1006583.

26. Dilthey AT, Jain C, Koren S, Phillippy AM. Strain-level metagenomic assignment and compositional estimation for long reads with MetaMaps. *Nat Commun*. 2019; doi: 10.1038/s41467-019-10934-2.

27. Jia B, Xuan L, Cai K, Hu Z, Ma L, Wei C. NeSSM: A Next-Generation Sequencing Simulator for Metagenomics. *PLoS One*. 2013; doi: 10.1371/journal.pone.0075448.

28. Fritz A, Hofmann P, Majda S, Dahms E, Dröge J, Fiedler J, et al.. CAMISIM: Simulating metagenomes and microbial communities. *Microbiome*. 2019; doi: 10.1186/s40168-019-0633-6.

29. O'Leary NA, Wright MW, Brister JR, Ciufo S, Haddad D, McVeigh R, et al.. Reference sequence (RefSeq) database at NCBI: Current status, taxonomic expansion, and functional annotation. *Nucleic Acids Res*. 2016; doi: 10.1093/nar/gkv1189.

30. Howe KL, Achuthan P, Allen J, Allen J, Alvarez-Jarreta J, Ridwan Amode M, et al.. Ensembl 2021. *Nucleic Acids Res*. 2021; doi: 10.1093/nar/gkaa942.

31. Kolmogorov M, Bickhart DM, Behsaz B, Gurevich A, Rayko M, Shin SB, et al.. metaFlye: scalable long-read metagenome assembly using repeat graphs. *Nat Methods*. 2020; doi: 10.1038/s41592-020-00971-x.

32. Patro R, Duggal G, Love MI, Irizarry RA, Kingsford C. Salmon provides fast and bias-aware quantification of transcript expression. *Nat Methods*. 2017; doi: 10.1038/nmeth.4197.

33. Martin S, Heavens D, Lan Y, Horsfield S, Clark MD, Leggett RM. Nanopore adaptive sampling: a tool for enrichment of low abundance species in metagenomic samples. *bioRxiv*. Cold Spring Harbor Laboratory; 2021;

34. Proctor LM, Creasy HH, Fettweis JM, Lloyd-Price J, Mahurkar A, Zhou W, et al.. The Integrative Human Microbiome Project. *Nature*. 2019; doi: 10.1038/s41586-019-1238-8.

35. Code Ocean Capsule: Characterization and simulation of metagenomic nanopore sequencing data with Meta-NanoSim https://doi.org/10.24433/CO.1273690.v1

36. Yang C, Lo T, Nip KM, Hafezqorani S, Warren RL, Birol I. Supporting data for "Characterization and simulation of metagenomic nanopore sequencing data with Meta-NanoSim" GigaScience Database. 2023. http://dx.doi.org/10.5524/102355

Click here to access/download;Figure;Fig1.pdf

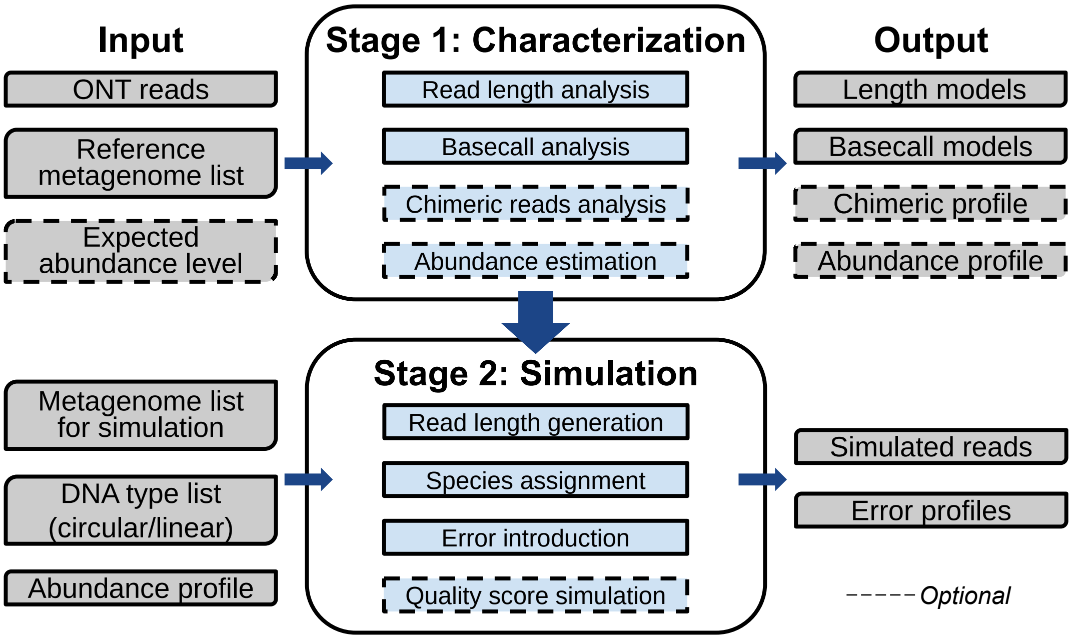

Figure 2 [Click here to access/download;Figure;Fig2.pdf]

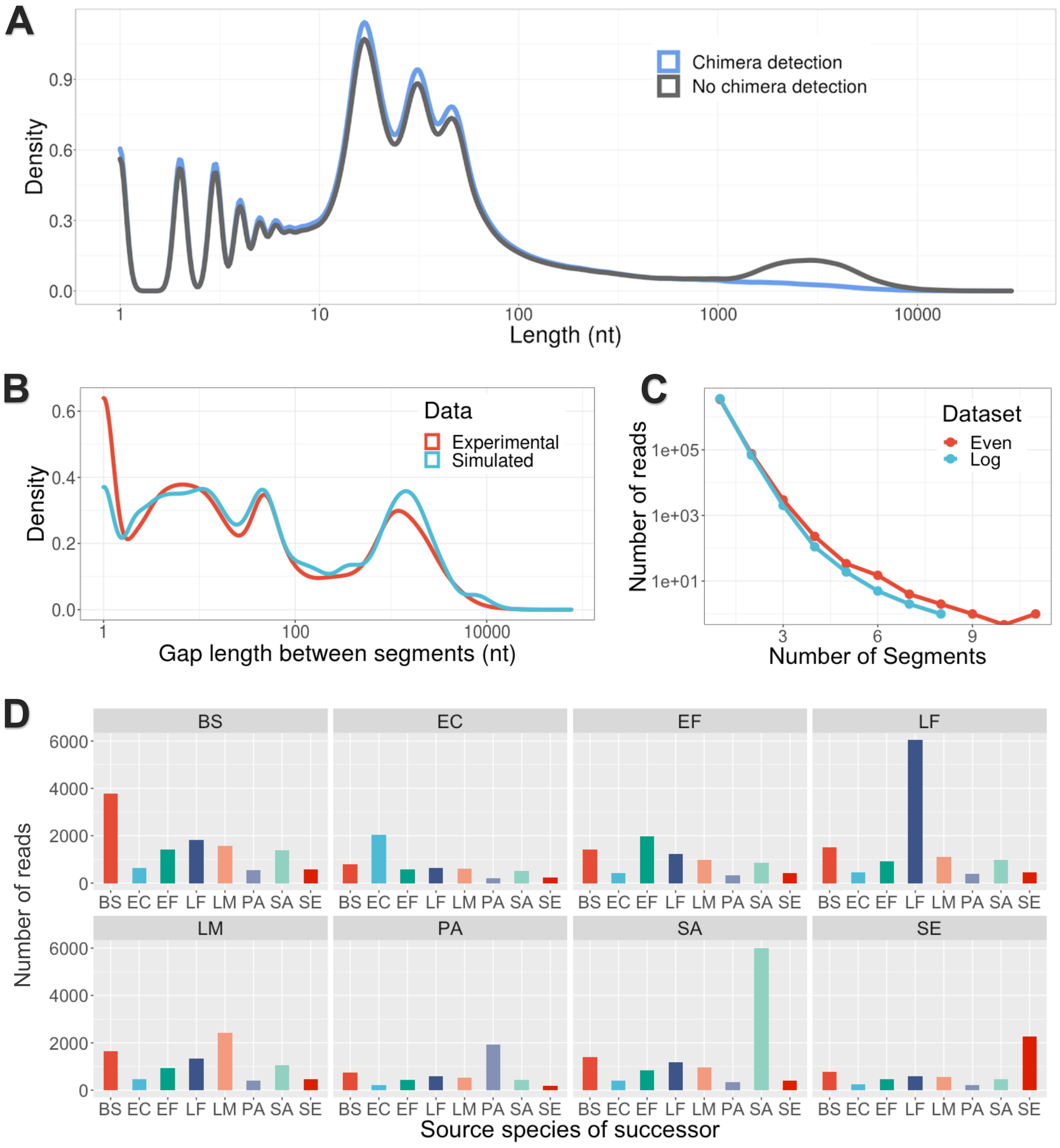

BS: *Bacillus subtilis* EC: *Escherichia coli* EF: *Enterococcus faecalis* LF: *Lactobacillus fermentum*
LM: *Listeria monocytogenes* PA: *Pseudomonas aeruginosa* SA: *Staphylococcus aureus* SE: *Salmonella enterica*

Figure 3

Click here to access/download;Figure;Fig3.pdf

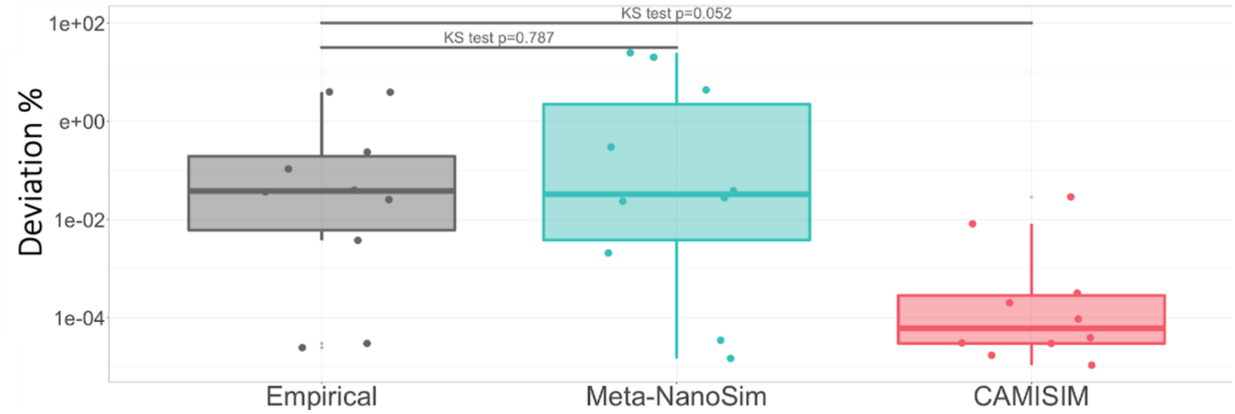

Figure 4

Click here to access/download;Figure;Fig4.pdf

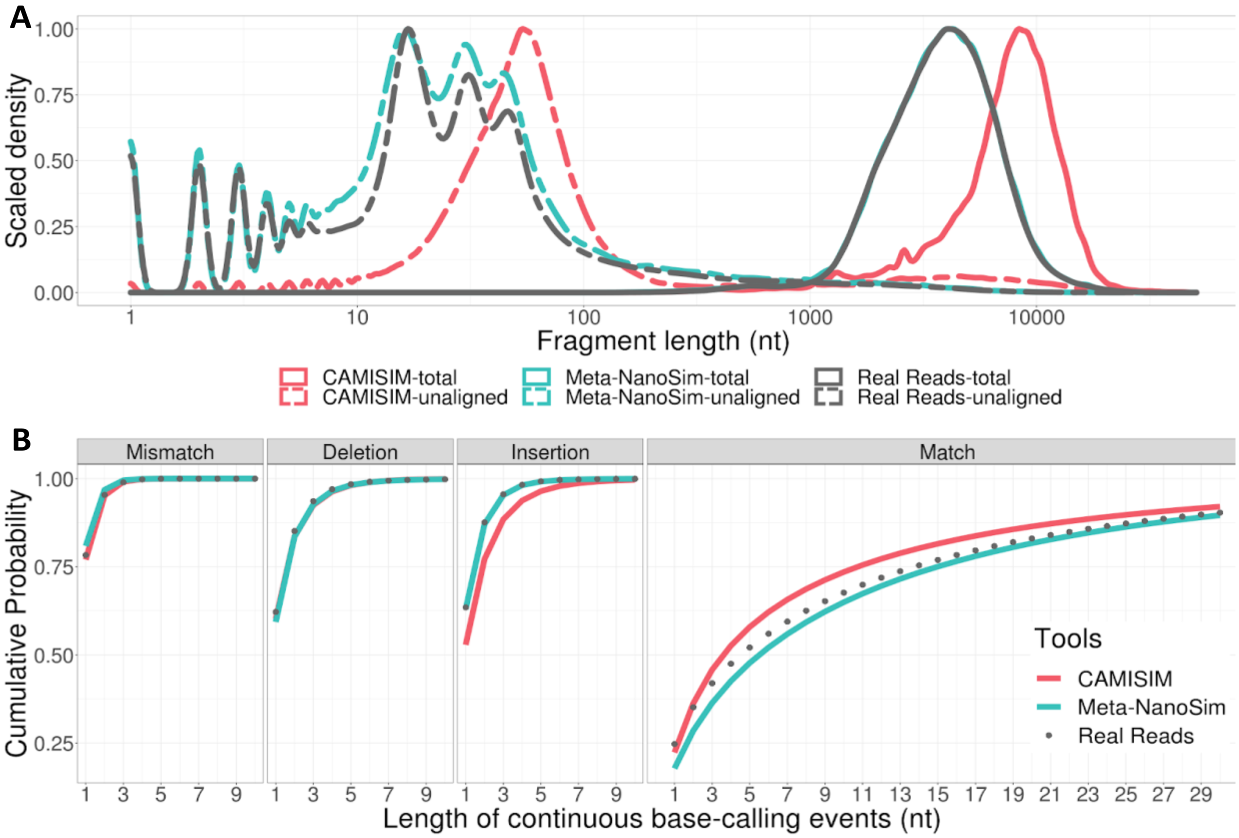

Figure 5

Click here to access/download;Figure;Fig5.pdf

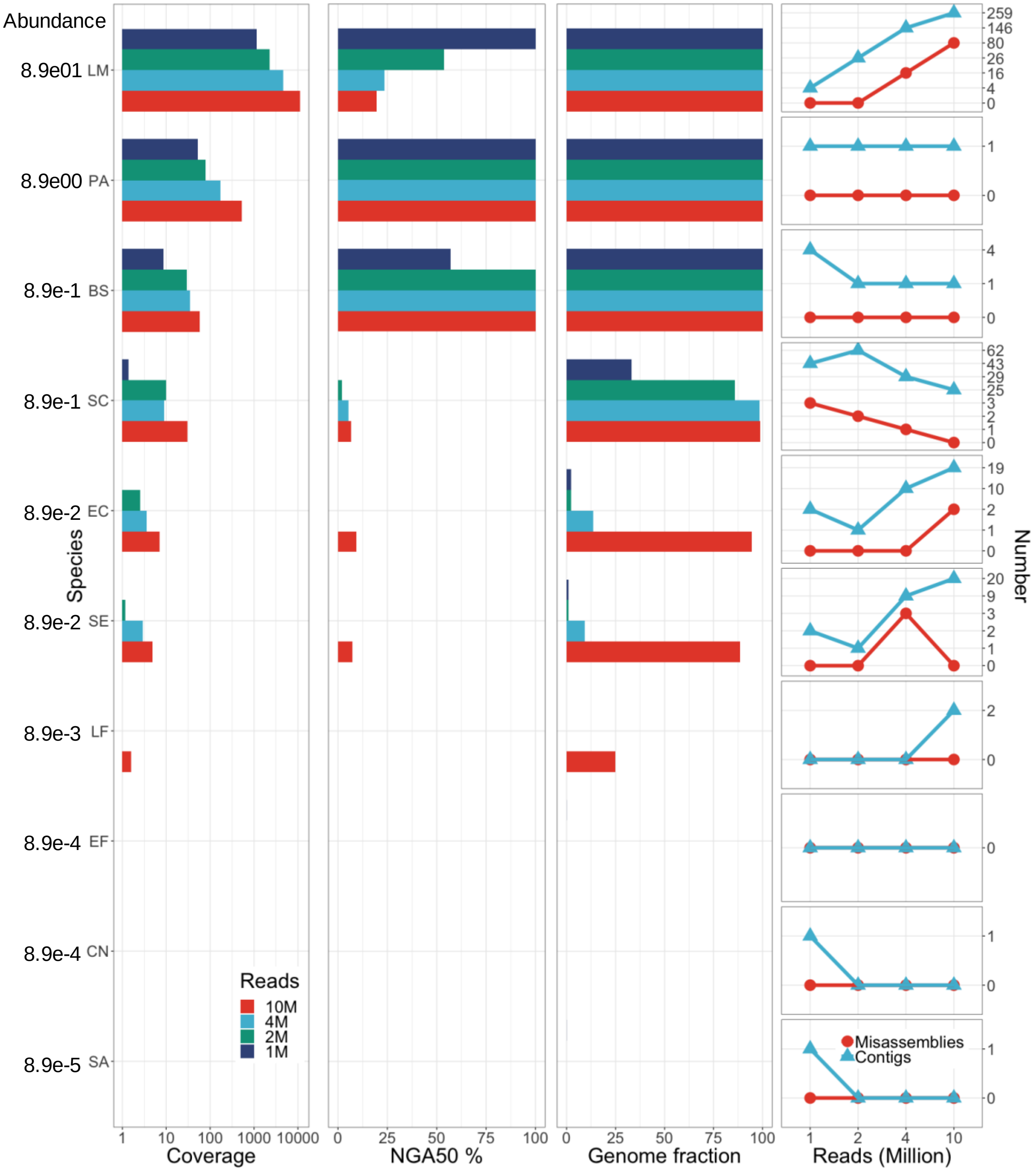

Additional File 1

Click here to access/download

Supplementary Material

meta-nanosim_gigascience_supplementary_material_revised.docx
