# Supplementary material for: Characterization and simulation of metagenomic nanopore sequencing data with Meta-NanoSim
Source: Gigascience. 2023 Mar 20;12:giad013. doi: 10.1093/gigascience/giad013 (PMC10025935; doi:10.1093/gigascience/giad013)
Supplement: giad013_Supplemental_File [file giad013_supplemental_file.docx]

**Additional File 1**

**Characterization and simulation of metagenomic nanopore sequencing data with Meta-NanoSim**

Chen Yang^1,2^, Theodora Lo^1,2^, Ka Ming Nip^1,2^, Saber Hafezqorani^1,2^, René L Warren^1^, Inanc Birol^1,3*^

1. Canada’s Michael Smith Genome Sciences Centre, BC Cancer, Vancouver, BC, Canada

2. Bioinformatics Graduate Program, University of British Columbia, Vancouver, BC, Canada

3. Department of Medical Genetics, University of British Columbia, Vancouver, BC, Canada

* Corresponding author

Table of contents

| **Supplementary Methods** | 3 |
| --- | --- |
| **Table S1 Statistical analysis of the abundance estimation on the Adp dataset** | 6 |
| **Table S2 MetaFlye assembly comparison** | 6 |
| **Fig. S1 Chimeric read detection and circularity check** | 7 |
| **Fig. S2 Average length distribution for each species.** | 8 |
| **Fig. S3 Abundance estimation comparison** | 9 |
| **Fig. S4 Performance of Meta-NanoSim in simulating one million reads** | 10 |
| **Fig. S5 Quality score distribution for one million Meta-NanoSim simulated reads** | 11 |

**Supplementary Methods**

**Datasets**

For training and testing meta-NanoSim, we used two publicly available ONT datasets generated on the GridION platform (8). ERR3152364 was sequenced from the Zymo CS Even ZRC190633 community, and ERR3152366 was sequenced from the Zymo CSII Log ZRC190842 community. Both communities contain eight species of bacteria and two species of fungi; the abundance levels are 12% for each bacterium and 2% for each fungus for the *Even* dataset; and the abundance levels for the *Log* dataset are shown in Fig. 3A. Both the raw signal and basecalled FASTQ files for these datasets are available at the European Nucleotide Archive (ENA), via the ERR identifiers mentioned above. To download these datasets, you need to visit the ENA website (https://www.ebi.ac.uk/ena/browser/home) and search for the identifiers. For example, for the Even dataset (ERR3152364) the following link contains the download information:

https://www.ebi.ac.uk/ena/browser/view/ERR3152364.

Please note that the reference genomes for *Cryptococcus neoformans* and *Saccharomyces cerevisiae* provided by Zymo community were draft assemblies and very fragmented, and hence, cannot be used for quantification. Therefore, for those two individual genomes, we downloaded RefSeq reference genomes and used them in our analysis. Reads were basecalled using Guppy v2.2.2 GPU basecaller with flip-flop configuration, and adapters were trimmed using Porechop v0.2.4. Reads were also split when internal adapters were found to eliminate false chimeric reads. Additionally, we used another publicly available ONT dataset, the *Adp* dataset, from project PRJEB44844. This dataset is composed of three libraries with different mean sequence length: ERR5897838, ERR5903399, and ERR5909878. Reads from this dataset were basecalled during the experiment on the GridION using the MinKNOW software. The abundance levels are 50% *A. xylosoxidans*, 25% *M. morganii*, 12% *L. richardii*, 6% *P. aeruginosa*, 4% *M. wisconsensis*, 2% *P. vulgaris* and 1% *S. dysgalactiae*.

The experimental dataset for naturally occurring microbial community is from the HMP project, human saliva sample SRS019120. The abundance table of the sample was downloaded from <https://hmpdacc.org/hmp/HMSCP/>. In the original file, there were 176 bacteria strains, and after removing the ones without reference genome in RefSeq, we compiled a list of 125 strains with their RefSeq reference genome ftp site. We computed the abundance levels based on the size of the reference genome and the estimated depth in the abundance file, and fed it into Meta-NanoSim for simulation. The simulated 10 million reads are uploaded to Zenodo for academic use: <https://doi.org/10.5281/zenodo.5712441>.

**Abundance estimation benchmarking**

For the Data Note estimation, we used the data provided in the paper where the dataset was first reported (8). For all other three methods, we first mapped the reads to the ZymoBIOMICS reference genome version 2 with Minimap 2.17-r941. To run Salmon, we used the --meta option and --noErrorModel to quantify the abundance for both datasets as suggested in the manual.

**Simulation and benchmarking**

We trained Meta-NanoSim on the mock community datasets with two options, --chimeric and --quantification (src/read_analysis.py metagenome -i input.fa -gl metagenome_list_for_training -t 12 -q -c), and then simulated one million reads with --chimeric (src/simulator.py metagenome -gl metagenome_list_for_simulation -a abundance_for_simulation.tsv -dl dna_type_list.tsv -c models/training -t 12 -c). The abundance levels simulated are the same as the *Log* dataset and *Adp* dataset, when respective metagenome were used for simulation. CAMISIM uses NanoSim as the engine for ONT read simulation. The pre-trained profile is hardcoded in CAMISIM, which is trained on an *E. coli* dataset with NanoSim version one. To run CAMSIM, we had to choose a most updated compatible version of NanoSim (V2.0.0) that reads the specific format of profiles. Then we mapped the simulated datasets to the reference genomes with Minimap 2.17-r941 to calculate the read length distributions and error distributions, and compared the result with the ones of the experimental data.

**Assembly benchmarking**

The assembly benchmarking was performed on a high-performance computing server with 128 CPUs and 1 TB memory. We simulated four sets of data with models trained on the *Log* dataset with default settings and each dataset contains 1, 2, 4, 10 million reads. Then we ran metaFlye 2.8.1-b1676 with options --meta, --plasmids, --threads 128 and -g 70m. Next we ran MetaQUAST v5.1.0rc1 with default settings to evaluate the quality of assemblies.

**Table S1 Statistical analysis of the abundance estimation on the Adp dataset.**

| **Tool** | **Algorithm** | | | | ***Adp* dataset** | | | |
| --- | --- | --- | --- | --- | --- | --- | --- | --- |
|  | **E** | **C** | **B** | **R** | **R^2^** | **Log R^2*^** | **Std*** | **PE** |
| **Meta-NanoSim** | √ | √ | √ |  | 0.8831 | 0.9752 | 0.3439 | 2.1020 |
|  |  | √ | √ |  | 0.8831 | 0.9752 | 0.3439 | 2.1020 |
|  | √ |  | √ |  | 0.8830 | 0.9752 | 0.3439 | 2.1037 |
|  |  |  | √ |  | 0.8830 | 0.9752 | 0.3439 | 2.1037 |
| **Salmon** | √ |  |  | √ | 0.1873 | 0.5045 | 1.5379 | 9.3848 |
| **MetaMaps** | √ |  |  | √ | 0.8281 | 0.9643 | 0.4130 | 2.0137 |

**R^2^:** R-squared, **Std**: standard deviation, **PE**: summation of percent error

**E**: EM algorithm, **B**: base-level quantification, **R**: read-level quantification, **C**: chimeric reads detection

* The expected and estimated abundances are log-transformed before calculating R-squared value and standard deviation.

**Table S2 Runtime and Maximum Resident Set Size comparison for the metaFlye assemblies.**

| **Dataset** | **1 Million** | **2 Million** | **4 Million** | **10 Million** |
| --- | --- | --- | --- | --- |
| **Runtime (hh:mm:ss)** | 1:14:35 | 2:15:33 | 3:34:31 | 7:00:18 |
| **Maximum Resident Set Size (GB)** | 24.61 | 40.39 | 76.38 | 212.19 |

|  |
| --- |
| **Fig. S1 Chimeric read detection and circularity check. A.** Chimeric read detection workflow. Given a read and all its alignments, compatible sub-alignments are computed and sorted to generate a list of compatible alignments. Then the best compatible alignment set is selected based on alignment score and alignment length. If the best compatible alignment set for a read contains two or more compatible alignments, it is considered as chimeric read. **B.** Circularity detection workflow. For circular genomes, if a read surpasses the start position, the alignment is split into a primary and a supplementary alignment. Meta-NanoSim finds these two alignments and treats them together as one. |

**
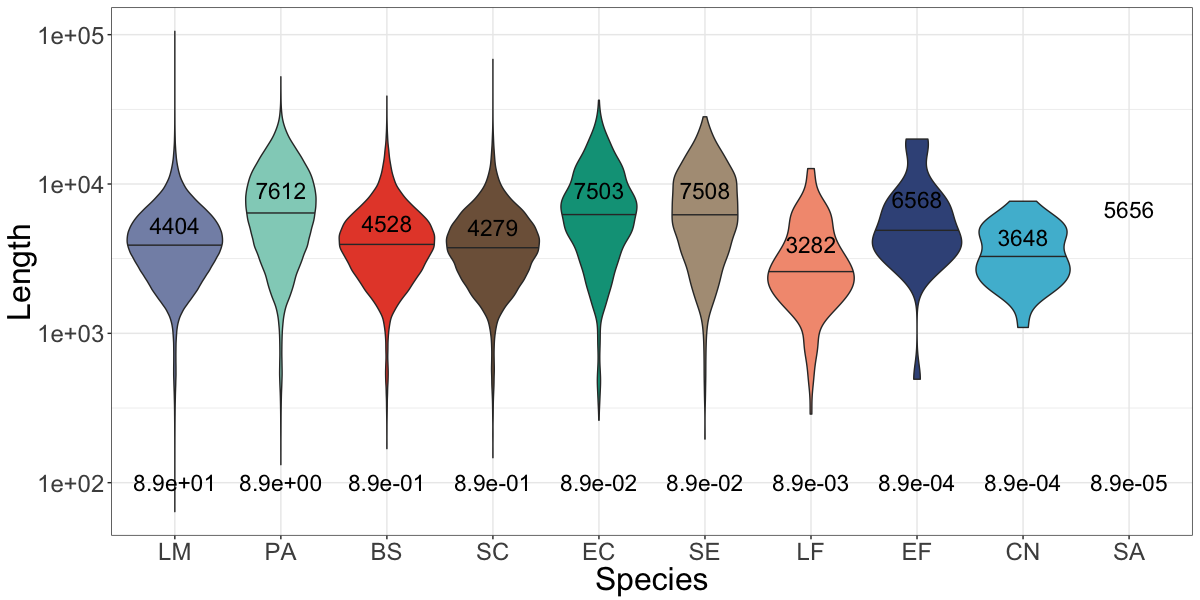
**

**Fig. S2 Average length distribution for each species.** This plot shows the average read lengths of uniquely aligned reads for each species. The horizontal line in each violin shows the median length and the number above it shows the mean length (in bp). The number at the bottom of each violin is the abundance level of each species. There are only two uniquely aligned reads for SA, so there is no violin to show. BS: *Bacillus subtilis*, CN: *Cryptococcus neoformans*, EC: *Escherichia coli*, EF: *Enterococcus faecalis*, LF: *Lactobacillus fermentum*, LM: *Listeria monocytogenes*, PA: *Pseudomonas aeruginosa*, SA: *Staphylococcus aureus*, SC: *Saccharomyces cerevisiae*, SE: *Salmonella enterica*.

| 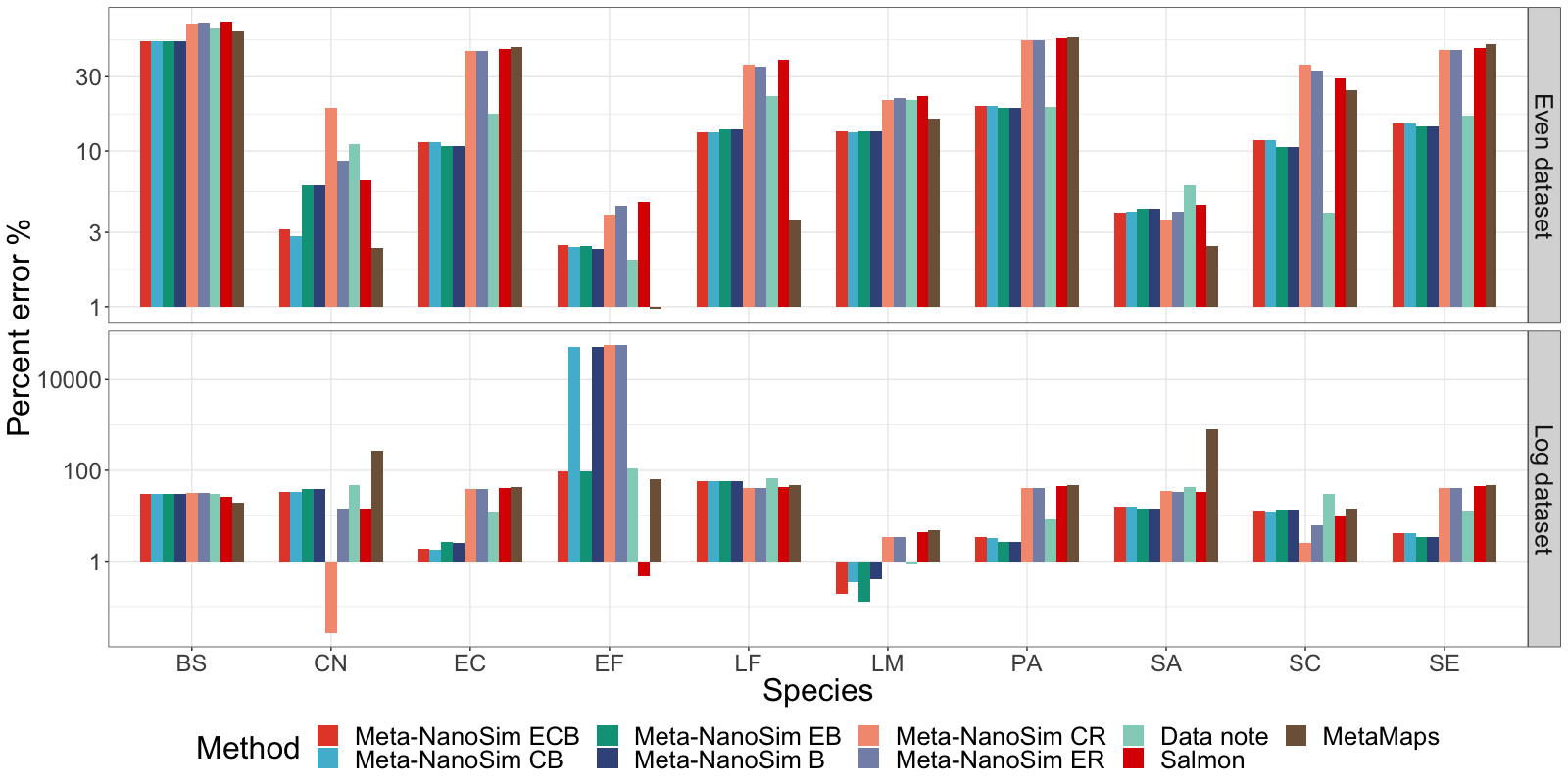 |
| --- |
| **Fig. S3 Abundance estimation comparison.** The metagenome abundance for *Even* and *Log* dataset is calculated using the nine methods, and percent error is calculated as a percentage deviation from the expected values. Meta-NanoSim ECB: EM algorithm + base-level quantification + chimeric reads detection. Meta-NanoSim EB: EM algorithm + base-level. Meta-NanoSim CB: chimeric reads detection + base-level quantification. Meta-Nanosim B: base-level quantification. Meta-NanoSim CR: chimeric reads detection + read-level quantification. Meta-NanoSim ER: EM algorithm + read-level quantification. Salmon: Salmon metagenomic quantification based on Minimap2 alignments. Data note: the abundance reported in the data releasing paper calculated based on Minimap2 alignments. BS: *Bacillus subtilis*, CN: *Cryptococcus neoformans*, EC: *Escherichia coli*, EF: *Enterococcus faecalis*, LF: *Lactobacillus fermentum*, LM: *Listeria monocytogenes*, PA: *Pseudomonas aeruginosa*, SA: *Staphylococcus aureus*, SC: *Saccharomyces cerevisiae*, SE: *Salmonella enterica*. |

**Fig. S4 Performance of Meta-NanoSim in simulating one million reads.** Meta-NanoSim used the *Log* dataset for simulating the seven-species metagenome (*Adp* dataset). Two samples were simulated at the same time, one million reads in each, with different abundance levels.

**A.** Comparison of read length distributions in the empirical vs. simulated reads (*x*-axis in logarithmic scale). Unaligned length represents the length of unaligned part of each aligned read. **B.** Cumulative probability function of the lengths of matches/errors in empirical and simulated reads.


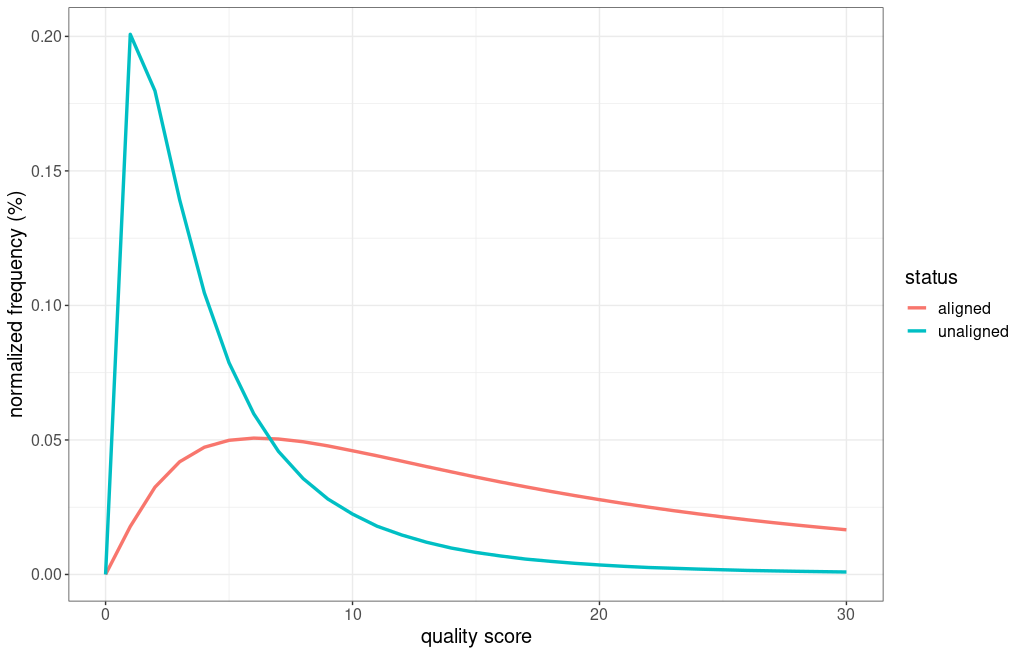


**Fig. S5 Phred quality score distribution for one million Meta-NanoSim simulated reads.** Meta-NanoSim used the *Log* dataset to simulate one million reads in FASTQ format. The horizontal axis corresponds to the phred quality scores in the reads, whereas the vertical axis corresponds to the normalized frequencies of quality scores. Frequencies are normalized for aligned and unaligned reads separately. The distribution of quality scores for aligned and unaligned reads are shown as red and teal lines, respectively. Overall, the aligned reads tend to have higher quality scores than the unaligned reads. This is expected because a higher quality score corresponds to a lower likelihood of sequencing base errors.
