# Supplementary material for: Characterization and simulation of metagenomic nanopore sequencing data with Meta-NanoSim
Source: Gigascience. 2023 Mar 20;12:giad013. doi: 10.1093/gigascience/giad013 (PMC10025935; doi:10.1093/gigascience/giad013)
Supplement: giad013_Response_to_Reviewer_Comments_Original_Submission [file giad013_response_to_reviewer_comments_original_submission.pdf]

Author's Response To Reviewer Comments

Close

Dear Dr. Zauner,

We would like to thank you for your consideration of our manuscript. The thorough and constructive feedback provided by you and the reviewers is greatly appreciated. In our revised submission, we have addressed the concerns raised, as outlined below in this letter, and have made the necessary revisions to the manuscript. Please note that the edited portions of the manuscript are highlighted in red, to make it easier to identify the changes compared to the original submission. In addition, our responses to the comments contain references to the line numbers of the changes made.

We would like to inform you that in accordance with your suggestion, we have included the CodeOcean capsule in the "availability" section and the DOI link in the bibliography, citing the capsule as per your suggestion. Additionally, we have deleted the sentence "Any restrictions to use by non-academics: Please contact the authors," from the availability section, since we use the GNU public license.

With regard to software registration in scientific resources, we would like to inform you that Meta-NanoSim has already been registered at the SciCrunch database and the RRID information has been included along with the FTP submission. All three NanoSim tools (NanoSim, Trans-NanoSim, and Meta-NanoSim) have also been registered at the bio.tools database with their corresponding identifiers. We have included this information in the "availability" section of the manuscript. Lastly, we would like to note that, as pertains to the workflowhub.eu, the Meta-NanoSim (and NanoSim) code has been implemented as a suite of modules, and there is no workflow being introduced in our manuscript.

Sincerely,

Saber Hafezqorani, Chen Yang, and Inanc Birol
Canada’s Michael Smith Genome Sciences Centre
British Columbia Cancer Agency

REVIEWER 1

• On line 64. Given the range of samples that can currently be sequenced using Nanopore sequencing and the recent focus on short reads as opposed to the previous highlight given to long reads, this statement is out-of-date. I would recommend describing instead the current range of sizes that can be sequenced via Nanopore.

Response: We clarified in the main text that ONT reads can have variable lengths. We also expanded on the N50 length metric definition. (Lines 63-65)

• How the authors would approach the different error rates namely of different types of flowcell (R9 vs R10). While I don't think this should be central to the development of the tool, I think this should be addressed in the manuscript in some way.

Response: Thank you for your comment about the error rates of different flowcells, which is an important point to consider as the nanopore technology matures. Indeed, Nanopore sequencing is an evolving technology and the error rates have improved tremendously since its emergence. Therefore, we agree that a simulator should be flexible to future advancements in this technology.

NanoSim is designed specifically to address this concern by learning and characterizing features of the input training data. Given a set of nanopore reads from a new flowcell (e.g. R10), the characterization

stage in NanoSim (the "read_analysis.py" script) can learn the features that are native to the platform. If users have any specific flowcell chemistry that they want to simulate, they can use the characterization module to train their own models for simulation.

For the convenience of our users, we have been providing ready-to-use pre-trained models for the past 5 years. We will continue to provide pre-trained models for more recent flowcells and respective chemistries in future releases of NanoSim.

We had initially addressed this concern when explaining the NanoSim workflow (Implementation section, Meta-NanoSim general design sub-section and Fig. 1) as well as in the Conclusion section. We have further expanded on this important point and added the following sentence for clarification (Lines 558-564):

"Considering the evolving Nanopore sequencing technology, with base accuracy improvements afforded by newer flowcells and updated chemistries, it is imperative to factor in those changes when simulating data with characteristics that are as close as possible to experimental data. The NanoSim suite of tools has this ability, which is accomplished by re-training new models on the latest available sequencing data. Pre-trained models are available, and will be supported along with future NanoSim releases to account for nanopore technology advancements."

• I would also have liked to see distributions of PHRED quality scores in the simulated reads in the analyses conducted in the manuscript.

Response: The simulated reads for our metagenome assembly benchmarking were generated in FASTA format. Therefore, quality scores do not contribute to the assembly performance of meta-Flye shown in Figure 5. We have investigated the distribution of quality scores in 1 million simulated reads based on the trained model for the Log dataset (Supplementary Figure S5). Overall, aligned reads tend to have higher quality scores than their unaligned counterparts. This is expected because a higher phred quality score corresponds to a lower likelihood of sequencing base errors (Lines 429-431).

REVIEWER 2:

• Line 166-168: the clause about structural variant is unclear to me, and perhaps to the reader. Please consider rephrasing.

Response: Thanks for your comment. We added clarity to the sentence and rephrased it as follows (Lines 167-171):

"They may arise because of sequencing artifacts, or may appear like structural variants when the source genome is absent from the reference metagenome. More specifically, sequencing reads may be mapped to a similar but structurally different genome (e.g. a different strain or subspecies), causing segments of the read to align to different regions of the reference genome."

• Line 209-210: I understand that the number of mapped reads is unadequate for abundance estimation of ONT data, but k-mers should not suffer from the same problem, shouldn't they? the number of k-mers matching to a genomic region (or a genome) will scale appropriately with read length. I have therefore a hard time understanding why k-mers are presented as problematic in the first sentence of the Abundance Estimation paragraph.

Response: There are two main reasons for this. First, as mentioned in the first sentence of that paragraph, the existing abundance estimation methods generally quantify the number of k-mers under the presumption that all reads have equal lengths, which is not a feature of ONT reads. Therefore, these algorithms originally designed for short reads are not directly applicable to ONT reads. Second, erroneous bases (due to mismatch or indel errors) in ONT reads would contribute to many low-multiplicity k-mers that do not belong to the reference. In other words, the k-mer multiplicities in long reads will very likely be an underestimate of the underlying microbial abundance levels. However, we acknowledge that this issue may be alleviated with improvements in the technology when the reads have much lower error rates.

• Line 379: What do you mean by "pronounced". Please consider rephrasing.

Response: Thanks for your comment, we rephrased the sentence as follows (Lines 380-383):

“The distribution of abundance deviations for experimental data and Meta-NanoSim simulated reads are statistically the same (Kolmogorov-Smirnov test p-value = 0.787), while the distributions for CAMISIM-simulated reads and experimental data are noticeably inconsistent, as CAMISIM does not provide this feature.”

• Line 438: geomes → genomes

Response: Thank you. We corrected the spelling. (Line number: 445)
We also corrected a couple of other spellings and added some punctuation marks. (Lines: 131, 166, 181, 217, 288, and 454)

• Figure 2: panel 2 should have the same theme as the other subplots of the figure

Response: We updated the subplot to account for this.

Software comments:

• I'd like to be able the examples present in the documentation out of the box: please add a link and instructions on how to download and unpack the zymo community

Response: The Supplemental Methods section in Additional File 1 describes the data analysed in this study. Thanks to your comment, we also added more information including instructions on how to download and access the Zymo mock community in the above-mentioned section.

• Speaking of the zymo community, why do Campylobacter and S. cerevisiae have a different path than the other genomes in the examples?

Response: The Zymo mock community does not contain Campylobacter spp. We assume you are referring to Cryptococcus neoformans. The reference genomes for Cryptococcus neoformans and S. cerevisiae provided by Zymo community are draft assemblies. We noticed that they are very fragmented and thus they cannot be used for quantification. Therefore, we downloaded their reference genomes from RefSeq, which resulted in the different paths. We clarified this in the Supplemental Methods - Datasets section (Additional File 1).

• If you plan to not update the pre-trained error models to a more recent version of scipy, please pin scipy 0.22.1 to the bioconda recipe, so that users can use pre-trained models out-of-the-box

Response: Thanks for flagging this technical detail. We assume you are referring to the scikit-learn package incompatibility issue but not scipy. In either case, please kindly note that we will include newly pre-trained models along with the next NanoSim release, which uses the latest versions of the required packages.

In the meantime, we also updated the NanoSim Github page with comprehensive information on how to avoid package incompatibility issues. We also updated the “requirements.txt” file used for installing NanoSim and relaxed unnecessary package version requirements, which should address this issue.

• Please make a new release of the software including pull request !67

Response: Thanks for pointing this out. Please note that pull request 167 is already solved and merged into the master branch. This will be automatically included in the next NanoSim release.

• In a future version of Nanosim, I urge you to consider gathering all scripts into subcommands (i.e. nanosim simulate [--params] instead of simulate.py [--params]. I realise this a big breaking change but it is good practise, and avoids polluting a user's PATH with many scripts. This change is in my opinion not required for the paper to be published, but something I'd like you to consider for a future release.

Response: Thanks for this great advice and suggestion. We will incorporate this in the next NanoSim release.

Close
