# Supplementary material for: Characterization and simulation of metagenomic nanopore sequencing data with Meta-NanoSim
Source: Gigascience. 2023 Mar 20;12:giad013. doi: 10.1093/gigascience/giad013 (PMC10025935; doi:10.1093/gigascience/giad013)
Supplement: giad013_Reviewer_1_Report_Original_Submission — Andre Rodrigues-Soares -- 11/26/2022 Reviewed [file giad013_reviewer_1_report_original_submission.pdf]

Reviewer Report

Title: Characterization and simulation of metagenomic nanopore sequencing data with Meta-NanoSim

Version: Original Submission **Date:** 11/26/2022

Reviewer name: Andre Rodrigues-Soares

Reviewer Comments to Author:

This manuscript is of very good quality - as such, my review is quite limited. I would like to congratulate the authors for the development of the software and its comprehensive and extensive benchmarking.On l. 64 - Given the range of samples that can currently be sequenced using Nanopore sequencing and the recent focus on short reads as opposed to the previous highlight given to long reads, this statement is out-of-date. I would recommend describing instead the current range of sizes that can be sequenced via Nanopore.After reading the manuscript, I am however, left to wonder how the authors would approach the different error rates namely of different types of flowcell (R9 vs R10). It can be assumed that error rates of R9 sequences might indeed average 10% as stated in the manuscript, but with the advent of R10 flowcells (more recently R10.4.1) and respective updated chemistries, the error rates have decreased significantly. One specific error rate of 10% as stated in the manuscript can't be assumed for the sequencing technology as a whole anymore. While I don't think this should be central to the development of the tool, I think this should be addressed in the manuscript in some way.I would also have liked to see distributions of PHRED quality scores in the simulated reads in the analyses conducted in the manuscript. Although the assembly and genome recovery statistics namely in Figure 4 indicate these should have the expected distributions, I would have liked to understand how quality scores are distributed in the generated reads. If the two issues above are addressed in the manuscript, I will be happy to recommend its publication.I have no further reviews to add as the manuscript covers all other factors I would think could be worrying regarding a tool simulating Nanopore metagenomic reads.

Level of Interest

Please indicate how interesting you found the manuscript: Choose an item.

Quality of Written English

Please indicate the quality of language in the manuscript: Choose an item.

Declaration of Competing Interests

Please complete a declaration of competing interests, considering the following questions:

- Have you in the past five years received reimbursements, fees, funding, or salary from an organisation that may in any way gain or lose financially from the publication of this manuscript, either now or in the future?

- Do you hold any stocks or shares in an organisation that may in any way gain or lose financially from the publication of this manuscript, either now or in the future?
- Do you hold or are you currently applying for any patents relating to the content of the manuscript?
- Have you received reimbursements, fees, funding, or salary from an organization that holds or has applied for patents relating to the content of the manuscript?
- Do you have any other financial competing interests?
- Do you have any non-financial competing interests in relation to this paper?

If you can answer no to all of the above, write 'I declare that I have no competing interests' below. If your reply is yes to any, please give details below.

I declare that I have no competing interests

I agree to the open peer review policy of the journal. I understand that my name will be included on my report to the authors and, if the manuscript is accepted for publication, my named report including any attachments I upload will be posted on the website along with the authors' responses. I agree for my report to be made available under an Open Access Creative Commons CC-BY license (http://creativecommons.org/licenses/by/4.0/). I understand that any comments which I do not wish to be included in my named report can be included as confidential comments to the editors, which will not be published.

Choose an item.

To further support our reviewers, we have joined with Publons, where you can gain additional credit to further highlight your hard work (see: https://publons.com/journal/530/gigascience). On publication of this paper, your review will be automatically added to Publons, you can then choose whether or not to claim your Publons credit. I understand this statement.

Yes Choose an item.
