# Supplementary material for: Characterization and simulation of metagenomic nanopore sequencing data with Meta-NanoSim
Source: Gigascience. 2023 Mar 20;12:giad013. doi: 10.1093/gigascience/giad013 (PMC10025935; doi:10.1093/gigascience/giad013)
Supplement: giad013_Reviewer_2_Report_Original_Submission — Hadrien GourlÃ -- 12/5/2022 Reviewed [file giad013_reviewer_2_report_original_submission.pdf]

Reviewer Report

Title: Characterization and simulation of metagenomic nanopore sequencing data with Meta-NanoSim

Version: Original Submission **Date:** 12/5/2022

Reviewer name: Hadrien GourlÃ©

Reviewer Comments to Author:

Thank you for a great piece of software and great article.A few minor points:l166-168: the clause about structural variant is unclear to me, and perhaps to the reader. Please consider rephrasing.l209-210: I understand that the number of mapped reads is unadequate for abundance estimation of ONT data, but k-mers should not suffer from the same problem, shouldn't they? the number of k-mers matching to a genomic region (or a genome) will scale appropriately with read length. I have therefore a hard time understanding why k-mers are presented as problematic in the first sentence of the Abundance Estimation paragraph.l379: What do you mean by "pronounced". Please consider rephrasing.l438: geomes -> genomesfigure 2: panel 2 should have the same theme as the other subplots of the figuresoftware comments:- I'd like to be able the examples present in the documentation out of the box: please add a link and instructions on how to download and unpack the zymo community- Speaking of the zymo community, why do Campylobacter and S. cerevisiae have a different path than the other genomes in the examples?- If you plan to not update the pre-trained error models to a more recent version of scipy, please pin scipy 0.22.1 to the bioconda recipe, so that users can use pre-trained models out-of-the-box- Please make a new release of the software including pull request !67- In a future version of Nanosim, I urge you to consider gathering all scripts into subcommands (i.e. nanosim simulate [--params] instead of simulate.py [--params]. I realise this a big breaking change but it is good practise, and avoids polluting a user's PATH with many scripts. This change is in my opinion not required for the paper to be published, but something I'd like you to consider for a future release.

Level of Interest

Please indicate how interesting you found the manuscript: Choose an item.

Quality of Written English

Please indicate the quality of language in the manuscript: Choose an item.

Declaration of Competing Interests

Please complete a declaration of competing interests, considering the following questions:

- Have you in the past five years received reimbursements, fees, funding, or salary from an organisation that may in any way gain or lose financially from the publication of this manuscript, either now or in the future?

- Do you hold any stocks or shares in an organisation that may in any way gain or lose financially from the publication of this manuscript, either now or in the future?
- Do you hold or are you currently applying for any patents relating to the content of the manuscript?
- Have you received reimbursements, fees, funding, or salary from an organization that holds or has applied for patents relating to the content of the manuscript?
- Do you have any other financial competing interests?
- Do you have any non-financial competing interests in relation to this paper?

If you can answer no to all of the above, write 'I declare that I have no competing interests' below. If your reply is yes to any, please give details below.

I declare that I have no competing interests

I agree to the open peer review policy of the journal. I understand that my name will be included on my report to the authors and, if the manuscript is accepted for publication, my named report including any attachments I upload will be posted on the website along with the authors' responses. I agree for my report to be made available under an Open Access Creative Commons CC-BY license (http://creativecommons.org/licenses/by/4.0/). I understand that any comments which I do not wish to be included in my named report can be included as confidential comments to the editors, which will not be published.

Choose an item.

To further support our reviewers, we have joined with Publons, where you can gain additional credit to further highlight your hard work (see: https://publons.com/journal/530/gigascience). On publication of this paper, your review will be automatically added to Publons, you can then choose whether or not to claim your Publons credit. I understand this statement.

Yes Choose an item.
